# Causal effect on the number of life years lost due to a specific event: average treatment effect and variable importance

**DOI:** 10.1007/s10985-026-09712-2

**Published:** 2026-07-31

**Authors:** Simon Christoffer Ziersen, Torben Martinussen

**Affiliations:** 1 Section of Biostatistics, University of Copenhagen, Copenhagen, Denmark; 2 Present Address: Department of Biostatistics, University of Oslo, Oslo, Norway

**Keywords:** Causal inference, Competing risks, Heterogeneity, Nonparametric inference, Number of life years lost, Variable importance measure

## Abstract

Competing risk is a common phenomenon when dealing with time-to-event outcomes in biostatistical applications. An attractive estimand in this setting is the “number of life-years lost due to a specific cause of death”. It provides a direct interpretation on the time-scale on which the data is observed. In this paper, we introduce the causal effect on the number of life years lost due to a specific event and give assumptions under which the average treatment effect (ATE) and the conditional average treatment effect (CATE) are identified from the observed data. Semiparametric estimators for the ATE and a partially linear projection of CATE, serving as a variable importance measure, are proposed. These estimators leverage machine learning for nuisance parameters and are model-agnostic, asymptotically normal, and efficient. We give conditions under which the estimators are asymptotically normal, and their performance is investigated in a simulation study. Lastly, the methods are implemented in a study concerning the response to different antidepressants using data from the Danish national registers.

## Introduction

Time-to-event outcomes are very common in the biomedical sciences, and often there is the additional complication of competing events. For example, Kessing et al. (2024) were interested in the time to non-response to different antidepressants for patients with a major depressive disorder. The event of interest was defined as a switch to or add-on of another antidepressant or antipsychotic medicine or readmission to a psychiatric hospital with a major depressive disorder, while admission to a psychiatric hospital with a higher order psychiatric diagnosis (bipolar disorder, schizophrenia or organic mental disorder) or death were competing risks. One purpose of that study was to compare a potential causal effect of treatment with the antidepressant Setraline versus treatment with another antidepressant, Escitalopram, and to determine whether such an effect was dependent on individual characteristics. With competing risk data, it is standard practice to target the average treatment effect (ATE) based on the cumulative incidence functions (CIF) associated with the competing events. Estimation of the ATE in this setting has been described by Ozenne et al. (2020) under working Cox models and logistic regression for the nuisance parameters, whereas Rytgaard et al. (2023) provided estimators of the ATE under data-adaptive nuisance estimators.

From a causal perspective, contrasts in counterfactual cause-specific CIFs correspond to *total effects* in the sense discussed by Young et al. (2020), which connects these quantities to the broader causal mediation framework of Robins and Greenland (1992). Such total effects rely on relatively weak identifying assumptions compared with notions of direct effects, since they do not attempt to disentangle treatment mechanisms operating through competing events.

In some scientific settings, investigators may be primarily interested in understanding treatment mechanisms operating through different event types. In such cases, alternative causal estimands may be considered. In particular, Stensrud et al. (2021, 2022) introduced the concept of separable effects, which aims to decompose treatment effects into components acting through distinct causal pathways associated with competing events. Martinussen and Stensrud (2023) generalized this concept to the continuous time setting. These estimands require stronger assumptions than the total effect we consider here.

In this paper we focus on a summary of total effects based on the concept of “years of life lost” proposed by Andersen (2013). Within a given time frame $[0,t^*]$ it aims, in the classical setting of competing causes of death, to decompose the years of life lost (in the considered time window) due to the specific causes of death. In our working example, where the first two years after the initiation of treatment were of most interest, it corresponds to healthy days lost before two years after the treatment initiation due to the primary cause and the competing causes. The resulting quantity can be viewed as a restricted mean - type measure defined relative to the fixed time horizon $t^*$, corresponding to an area-under-the-curve summary of the cause-specific cumulative incidence function over $[0,t^*]$. As such, it provides a scalar summary of the total effect captured by the cause-specific cumulative incidence function. While reporting the entire cumulative incidence curve may reveal potential time variation in effects, the restricted mean–type summary offers a concise and interpretable measure expressed directly in time units. In this paper, we introduce the ATE based on this quantity - number of life years lost due to a specific event. We provide an estimator of the ATE based on semiparametric efficiency theory, allowing for the use of machine learning methods for nuisance parameter estimation without relying on model-specifications. We further give assumptions on the nuisance estimators under which the proposed ATE estimator is asymptotically normal and nonparametric locally efficient.

Andersen et al. (2017) provide a general procedure for estimating the ATE with censored data, where the ATE based on the number of life years lost due to a specific event serves as an example. Their estimator is based on pseudo-observations and its asymptotic properties in the presence of covariate dependent censoring are derived in Overgaard et al. (2019). Specifically, their estimator relies on a correctly specified parametric model for the conditional average treatment effect along with a correctly specified model for the censoring distribution. In contrast, our estimator does not rely on such parametric model specifications, and it allows for estimation of the involved nuisance parameters based on machine learning, while still providing inference for the ATE estimate.

Importantly, we also extend the treatment effect variable importance measure given in Ziersen and Martinussen (2025) as a best partially linear projection of the conditional average treatment effect (CATE). The projection parameter provides an attractive measure of potential treatment effect heterogeneity through a given covariate - this is of great interest in many real applications as the one we present in Sect. 6 on evaluating antidepressant treatment effects as mentioned above. We develop an estimator of this estimand that is also semiparametrically efficient and allows for machine learning methods to estimate nuisance parameters. The estimator admits an asymptotic normal distribution which defines a test of treatment effect heterogeneity of a given covariate.

In Sect. 2 we state the notation and setup used in the paper, and in Sect. 3 we define two target parameters. Section 4 gives the efficient influence functions for the two target parameters and utilizes these to construct cross-fitted one-step estimators. The asymptotic distributions of the estimators are then proved under high level assumptions on the nuisance parameter estimates. In Sect. 5, the finite sample performance of the proposed estimators are investigated in a simulation study and in Sect. 6 we apply the estimators to a study on treatment response to different antidepressants based on data from the Danish national registers (Kessing et al. 2024). Section 7 concludes the paper with some final remarks.

## Notation and setup

We consider a time-to-event setting with competing risks. Let *T* be the time to event and $\varDelta \in \{1,2\}$ the event indicator for two competing events. Let *X* be a *d*-dimensional vector of covariates, and let *A* denote the baseline treatment indicator. We enforce censoring through a censoring time *C*, such that the observed event time is ${\tilde{T}} = T \wedge C$ and the observed event indicator is ${\tilde{\varDelta }} = \mathbb {1}(C\ge T)\varDelta$. Our observed data, ${\mathcal {O}}$, is given by *n* i.i.d. copies of $O = ({\tilde{T}}, {\tilde{\varDelta }}, A, X)\sim P_0$, where $P_0 \in {\mathcal {M}}$, with ${\mathcal {M}}$ being a nonparametric model.

We introduce the conditional cause-specific hazard functions, $\lambda _{0, j}(t\mid a, x)$, for the *j*’th cause, *j=1,2*, and let $\lambda _{0,c}(t\mid a,x)$ denote the censoring hazard function. We let $\varLambda _{0, j}(t\mid a, x) = \int _0^t \lambda _{0, j}(s\mid a, x)\textrm{d}s$ and $\varLambda _{0, C}(t\mid a, x) = \int _0^t \lambda _{0, C}(s\mid a, x) \textrm{d}s$ denote the corresponding cumulative hazard functions. We denote $S(t\mid a, x) = \exp \{-\varLambda _1(t\mid a,x) - \varLambda _2(t\mid a,x)\}$ the survival function, and $\pi _0(a\mid x) = P_0(A = a\mid X=x)$ the conditional distribution of *A* given *X*. Let $N_j(t) = \mathbb {1}({\tilde{T}} \le t, {\tilde{\varDelta }} = j)$ denote the observed counting process for the *j*’th event and let $M_j(t\mid a,x)$ denote the corresponding martingale conditional on *A=a* and *X=x* such that $M_j(\textrm{d}t\mid a,x) = N_j(\textrm{d}t) - \mathbb {1}({\tilde{T}}\ge t)\varLambda _{0,j}(\textrm{d}t\mid a,x)$.

We use the notation $Pf = \int f \textrm{d}P$ and ${\mathbb {P}}_n f = \sum _{i=1}^n f(O_i)$, and $E_0 \{ f(O) \} = \int f \textrm{d}P_0$ is the expectation of *f*(*O*) under the true data generating distribution. Throughout, for a given function ${\hat{f}}$, which might be estimated from data, all expectations, $P{\hat{f}}$, considers the function ${\hat{f}}$ fixed, i.e. the expectation is not taken with respect to the randomness in ${\hat{f}}$. Finally, $\left\Vert \cdot \right\Vert$ denotes the $L_2(P)$-norm, such that $\left\Vert f\right\Vert = \left( \int f^2 \textrm{d}P \right) ^{1/2}$.

## Causal estimand and nuisance parameters

Define $Y_j(t^*)=(t^*-T\wedge t^*)\mathbb {1}(\varDelta = j)$. Then ${{\,\mathrm{\textrm{E}}\,}}Y_j = \int _0^tF_j(s)\textrm{d}s$, where $F_j$ is the cumulative incidence function for event *j*. In the following we will motivate the choice of $Y_j$ as an outcome of interest, and use it to construct counterfactuals.

Inspired by Andersen (2013), we introduce

$$L_0(0,t^*|a,x)=t^*-\int _0^{t^*}S(u|a,x)du$$

for a given time-horizon $[0, t^*]$, which can be interpreted as the expected number of years lost before time $t^*$ in strata (*a*, *x*). As Andersen (2013) shows, this quantity can be decomposed naturally into

$$L_0(0,t^*|a,x)=L_1(0,t^*|a,x)+L_2(0,t^*|a,x)$$

where

$$L_j(0,t^*|a,x)=\int _0^{t^*}F_j(u|a,x)du,\quad j=1,2,$$

can be interpreted as number of years lost “due to cause *j*” (Andersen 2013), with $F_j$ being the *j*th cumulative incidence function given *A=a* and *X=x*, i.e. $F_j(t\mid a, x) = \int _0^t S(s\mid a, x)\textrm{d}\varLambda _j(s\mid a, x)$. We now introduce the counterfactual $Y^a_j(t^*) = (t^* - T^a \wedge t^*)\mathbb {1}(\varDelta ^a=j)$ for *a=0,1*, where $T^a$ and $\varDelta ^a$ are the time to event and event indicator, respectively, under treatment *a*. Then $Y^a_j(t^*)$ is the number of life-years lost due to event *j* before time $t^*$ under treatment *a*. We define the *j*’th-specific ATE as

$$E_0\{Y_j^1(t^*) - Y_j^0(t^*)\}$$

and the CATE is

$$E_0(Y_j^1(t^*) - Y_j^0(t^*)\mid X=x).$$

In order to identify the ATE and CATE from the observed data we need the following assumptions:

### Assumption A (Identification)

1. (Consistency) $$Y_j(t^*) = AY_j^1(t^*) + (1-A)Y_j^0(t^*)$$ a.s.
2. (Exchangeability) $$Y^a_j(t^*) \perp \!\!\! \perp A \mid X, \ a=0,1.$$  
3. (Positivity) $$\pi (a\mid x)P_0(C>t| A=a, X=x)P_0(T> t| A=a,X=x)$$$$> \eta> 0, \ \forall (t,x) \in [0,t^*]\times {\mathcal {X}}, \ a=0,1$$.
4. (Independent censoring) $$T\perp \!\!\! \perp C \mid A, X$$.

Define

$$\tau _j(x;t^*) \equiv L_j(0,t^*|1,x)- L_j(0,t^*|0,x),\quad j=1,2.$$

In Appendix B, we show that the CATE function is identified in the observed data because

$$E_0(Y_j^1(t^*) - Y_j^0(t^*)\mid X=x) = \tau _j(x;t^*),$$

and the average treatment effect as

$$E_0\{Y_j^1(t^*) - Y_j^0(t^*)\} = E_0\{\tau _j(X;t^*)\}$$

under assumption A. Going forward we drop the dependence of $t^*$ and write $\tau _j(x) = \tau _j(x;t^*)$ to ease notation. Based on the identification results, we define two target parameters as mappings from the model ${\mathcal {M}}$ on the observed data to the reals. The first parameter is the *j*-specific average treatment effect, defined as the mapping $\psi _j : {\mathcal {M}}\rightarrow {\mathbb {R}}$, where

$$\psi _j(P) = E\{L_j(0,t^*|1,X)- L_j(0,t^*|0,X) \}.$$

The second parameter is defined as a variable importance measure of the *l*’th covariate on $\tau _j(x)$ based on the best partially linear projection given in Ziersen and Martinussen (2025). It is defined as the mapping $\varOmega _j^l: {\mathcal {M}}\rightarrow {\mathbb {R}}$ with

$$\varOmega _j^l(P) = \frac{E\{{{\,\mathrm{\textrm{cov}}\,}}(X_l, \tau _j(X) \mid X_{-l})\}}{E\{{{\,\mathrm{\textrm{var}}\,}}(X_l\mid X_{-l})\}},$$

where $X_{-l}$ denotes the covariates indexed by $\{1, \ldots , d\} \setminus \{l\}$. The parameter $\varOmega _j^l$ can be viewed as a weighted average of the conditional covariance of the CATE and the covariate $X_l$ given the rest of the covariates. The parameter is dependent on the scale of the covariate in question, and when assessing the importance of different covariates it is not the estimate of the parameter that determines if a covariate is deemed of variable importance, but rather the test of $H_0:\varOmega _j^l = 0$, since $\varOmega _j^l$ is zero if there is no heterogeneity explained by $X_l$, as is seen by the following observation. The parameter can be derived as the least-squares projection of the CATE onto the partially linear model. To see this, let

$$\tau _j(x) = \beta X_l + w(X_{-l}) + R(X_l, X_{-l})$$

and define

$$(\beta ^*, w^*) = {\mathop {\mathrm{arg\,min}}\limits _{\beta , w}}{{\,\mathrm{\textrm{E}}\,}}\{ \tau _j(X) - \beta X_l - w(X_{-l})\}^2 = {\mathop {\mathrm{arg\,min}}\limits _{\beta , w}}{{\,\mathrm{\textrm{E}}\,}}R(X_l,X_{-l})^2,$$

where $\beta \in {\mathbb {R}}$ and *w* is some measurable function with finite variance. Then $\beta ^* = \varOmega _j^l(P)$ (Ziersen and Martinussen 2025). Note that the partially linear model is not assumed to hold, but in the case of no heterogeneity, i.e. *β =R=0*, we have $\varOmega _j^l=0$. Hence a test of $H:\varOmega _j^l=0$ is conservative in the sense that $\varOmega _j^l$ is identically zero when there is no heterogeneity, but it may also be zero in the case of actual heterogeneity through $X_l$ via e.g. an interaction between $X_l$ and $X_{l'}$, *l≠ l'* (i.e. when *R≠ 0*).

From the definition above $\varOmega _j^l$ can be interpreted as a regression coefficient when the partially linear model holds for $\tau _j$ (and not only in the case of no heterogeneity). But since this is rarely known in practice, caution should be taken in interpreting $\varOmega _j^l$ directly. For more details and discussion, see Ziersen and Martinussen (2025).

## Nonparametric estimation and inference

We base the estimation of the two target parameters, $\psi _j(P)$ and $\varOmega _j^l(P)$, on semiparametric efficiency theory (Bickel et al. 1993; van der Vaart 2000, Ch. 25). A key ingredient is the efficient influence function (EIF), which establishes the information bound related to the given target parameter and is used to construct estimators with desirable properties, such as robustness and efficiency, for said parameter. We refer to Kennedy (2022) for an introduction to these methods, which we briefly outline in Appendix A in relation to our target parameters.

### Average treatment effect

To derive an estimator for the ATE we first derive its EIF. We define the nuisance parameter $\nu = (\varLambda _1, \varLambda _2, \varLambda _c, \pi )$. The EIF is then given in the following lemma.

#### Lemma 1

For a given *j*, the efficient influence function of $\psi _j(P)$ is given by

$$\begin{aligned} {\tilde{\psi }}_{\psi _j}(O;\nu ) = \varphi _j(O;\nu ) - \psi _j(P), \end{aligned}$$

where $\varphi _j(\nu ;O)$ is a real-valued function defined on the sample space of *O* at a given value of *ν* with

1 $$\begin{aligned} \varphi _j(O;\nu ) = \tau _j(X) + \left( \frac{\mathbb{1}(A=1)}{\pi (1\mid X)} - \frac{\mathbb{1}(A=0)}{\pi (0\mid X)}\right) \biggl \{\sum _{i=1,2} \int _0^{t^*} \frac{H_{ij}(s,t^*\mid A, X)}{S_C(s\mid A, X)} \textrm{d}M_i(s\mid A, X)\biggr \} \end{aligned}$$

where

2 $$\begin{aligned} H_{ij}(s,t\mid a,x) = \int _s^t \mathbb {1}(i=j) + \frac{F_j(s\mid a, x) - F_j(u\mid a, x)}{S(s\mid a, x)} \textrm{d}u. \end{aligned}$$

#### Proof

See Appendix C. *□*

The function $\varphi _j(O;\nu )$ is the uncentered EIF of the ATE and will also appear in the development of an estimator for the best partially linear projection in Sect. 4.2. As the EIF of the ATE is linear in the target parameter, the *one-step* estimator for $\psi _j$ reduces to

3 $$\begin{aligned} {\hat{\psi }}_j^{OS} = {\mathbb {P}}_n \varphi _j(\cdot ;{\hat{\nu }}), \end{aligned}$$

where ${\hat{\nu }}$ is an estimate of *ν*. In Appendix A, we detail the construction of the so-called *cross-fitted* one-step estimator, which is used to alleviate a ceratin Donsker class condition, thus allowing for more flexible nuisance estimators. It is a sample-splitted version of the one-step estimator, where data is split into *K* folds ${\mathcal {V}}_k$, *k=1… K*. For each fold *k*, a nuisance estimator ${\hat{\nu }}_{-k}$ is obtained from the *k*’th leave-out sample ${\mathcal {V}}_{-k}$, excluding the *k*’th fold, and the average in Eq. (3) is taken over observations in the *k*’th fold. The cross-fitted estimator, denoted ${\hat{\psi }}_j^{CF}$, is then obtained as a weighted (according to the number of observations in each fold) average of the *K* estimates.

In order to derive asymptotic results for ${\hat{\psi }}_j^{CF}$ we need a set of assumptions on the nuisance estimators. The assumptions below are stated for a general nuisance estimator ${\hat{\nu }}$, but in applications to cross-fitted estimators, they are assumed to hold for each leave-out sample ${\mathcal {V}}_{-k}$. We will be explicit about this when stating results on the obtained estimators, but it is left out of assumption B for notational convenience.

#### Assumption B

The nuisance estimator ${\hat{\nu }}$ satisfies the following conditions

1. There exist a real-valued constant *η>0* such that ${\hat{S}}(t\mid a,x)> \eta$, $S(t\mid a,x)> \eta$, ${\hat{S}}_C(t\mid a,x)> \eta$, $S_C(t\mid a,x)> \eta$, ${\hat{\pi }}(a \mid x)> \eta$, $\pi (a \mid x)> \eta$ for all $(t,a,x) \in [0,t^*] \times \{0,1\} \times {\mathcal {X}}$.
2. For *a=0,1*, it holds that
  $$\begin{aligned}&{{\,\mathrm{\textrm{E}}\,}}_0\left\{ \sum _{i=1,2} \int _0^{t^*} S(s\mid a, X) {\hat{H}}_{ij}(s, t^*\mid a, X) \right. \\&\left. \phantom {{{\,\mathrm{\textrm{E}}\,}}\sum _{i=1,2} }\times \left( 1 - \frac{\pi (a\mid X)S_C(s\mid a, X)}{{\hat{\pi }}(a\mid X){\hat{S}}_C(s\mid a, X)} \right) \textrm{d}\left[ {\hat{\varLambda }}_i(s\mid a, X) - \varLambda _i(s\mid a, X) \right] \right\} = o_p(n^{-1/2}). \end{aligned}$$
3. For *a=0,1*, it holds that
  $$\begin{aligned} {{\,\mathrm{\textrm{E}}\,}}_0\left\{ \sup _{s\le t^*} \left| {\hat{\varLambda }}_1(s\mid a, X) - \varLambda _{1,0}(s\mid a, X)\right| \right\} ^2&= o_p(1) \\ {{\,\mathrm{\textrm{E}}\,}}_0\left\{ \sup _{s\le t^*} \left| {\hat{\varLambda }}_2(s\mid a, X) - \varLambda _{2,0}(s\mid a, X)\right| \right\} ^2&= o_p(1) \\ {{\,\mathrm{\textrm{E}}\,}}_0\left\{ \sup _{s\le t^*} \left| {\hat{\varLambda }}_c(s\mid a, X) - \varLambda _{c,0}(s\mid a, X)\right| \right\} ^2&= o_p(1) \\ {{\,\mathrm{\textrm{E}}\,}}_0\left\{ {\hat{\pi }}(a\mid X) - \pi _{0}(a\mid X)\right\} ^2&= o_p(1) \end{aligned}$$

Assumption B1 is the usual positivity assumption found through out the causal inference literature (for examples with censored data, see e.g. Westling et al. (2024), Rytgaard et al. (2023)). Whereas assumption A3 relates to the true data generating mechanism, assumption B1 extends to the estimators as well. We note that employing cross-fitting in relatively small sample sizes can sometimes lead to practical positivity-violations, when a “rare” covariate lies in ${\mathcal {V}}_k$ but not in ${\mathcal {V}}_{-k}$. For B3 to hold, all nuisance estimators must be consistent, and for the hazard estimators this amounts to uniform consistency. This requirement suggest the use of flexible learners for nuisance estimation. Assumption B2 reflects the dobule robustness that is common for one-step estimators. In Lemma D1 in the Appendix it is shown that the so-called remainder term, related to a decomposition of the estimator, takes this form. It is sometimes referred to as a second order remainder term, when it can be shown to hold if each of the nuisance estimators converge on $n^{-1/4}$-rate in $L_2(P)$-norm. If one considers cumulative hazard estimators that are absolute continuous, the result can be obtained from a simple application of the Cauchy-Schwarz inequality (for examples involving Highly Adaptive Lasso, see Munch et al. (2024), Rytgaard et al. (2022, 2023), but this exclude many commonly used cumulative hazard estimators such as any Breslow-type estimators. For such estimators, other techniques are required (see e.g. Munch et al. 2023). We expect nonetheless that the double robustness is obtained for most reasonable estimators, and in the later simulation studies, this will be exemplified by the use of random survival forests (Ishwaran et al. 2008).

Next follows our main result for the cross-fitted ATE estimator:

#### Theorem 1

Assume that the nuisance estimators ${\hat{\nu }}_{-k}$, *k=1… K*, follow assumption B for each *k*. Then the cross-fitted estimator for a given *j*, ${\hat{\psi }}_j^{CF}$, is asymptotically linear with influence function given by ${\tilde{\psi }}_{\psi _j}$ and hence

$$\sqrt{n}\left( {\hat{\psi }}_j^{CF} - \psi _j \right) \overset{d}{\rightarrow }\ {\mathcal {N}}(0, P{\tilde{\psi }}_{\psi _j}(\cdot , \nu _0)^2).$$

#### Proof

See Appendix D. *□*

In practice, the variance of the influence function is estimated by the cross-fitted plug-in estimator ${\hat{\sigma }}_{\psi _j}^{2,CF}$, which we detail in Appendix A. The standard error of ${\hat{\psi }}_j^{CF}$ is then given by $\sqrt{{\hat{\sigma }}_{\psi _j}^{2,CF}/n}$.

### Best partially linear projection

Estimation of $\varOmega _j^l(P)$ follows the same overall strategy, i.e., construct an asymptotically linear estimator using semiparametric theory. The difference now is that the target parameter is a ratio of two parameters which can both be written as a map from ${\mathcal {M}}$ to the reals. If we construct asymptotically linear estimators for both parameters in the ratio, separately, then the ratio of the estimators will also be asymptotically linear. Furthermore, if each estimator in the ratio has their respective EIF as their influence function, the ratio of the estimators will have its EIF as its influence, by the functional delta method (van der Vaart 2000, Ch. 25.7). In the following we will extend the approach of Ziersen and Martinussen (2025) to the CATE function defined by the number of life years lost, $\tau _j(x)$, for a given time-horizon $[0,t^*]$, where each of the parameters in the ratio of $\varOmega _j^l$ is estimated separately. We start by calculating the EIF for the relevant parameters.

#### Lemma 2

For a fixed *l* and *j*, define the mappings $\varGamma _j^l:{\mathcal {M}}\rightarrow {\mathbb {R}}$ and $\chi _j^l:{\mathcal {M}}\rightarrow {\mathbb {R}}$ as

$$\varGamma _j^l(P) = E\{{{\,\mathrm{\textrm{cov}}\,}}(X_l, \tau _j(X) \mid X_{-l})\}$$

and

$$\chi ^l(P) = E\{{{\,\mathrm{\textrm{var}}\,}}(X_l\mid X_{-l})\}$$

such that $\varOmega _j^l(P) = \frac{\varGamma _j^l(P)}{\chi ^l(P)}$. The efficient influence functions of $\varGamma _j^l(P)$, $\chi ^l(P)$ and $\varOmega _j^l(P)$, respectively, are given by

4 $$\begin{aligned} {\tilde{\psi }}_{\varGamma _j^l}(O;P)&= [\varphi _j(O;\nu ) - {{\,\mathrm{\textrm{E}}\,}}(\tau _j(X)\mid X_{-l})][X_l - {{\,\mathrm{\textrm{E}}\,}}(X_l\mid X_{-l})] - \varGamma _j^l(P), \end{aligned}$$

5 $$\begin{aligned} {\tilde{\psi }}_{\chi ^l}(O;P)&= [X_l - {{\,\mathrm{\textrm{E}}\,}}(X_l\mid X_{-l})]^2 - \chi ^l(P), \end{aligned}$$

6 $$\begin{aligned} {\tilde{\psi }}_{\varOmega _j^l}(O;P)&= \frac{1}{\chi ^l(P)}\left( {\tilde{\psi }}_{\varGamma _j^l}(O;P) - \varOmega _j^l(P) {\tilde{\psi }}_{\chi ^l}(O;P) \right) . \end{aligned}$$

#### Proof

See Appendix C. *□*

The EIF’s above depend explicitly on the conditional distribution of $X_l$ given $X_{-l}$ through $E(X_l\mid X_{-l})$ and $E(\tau _j(X)\mid X_{-l})$, so to express them as mappings of the nuisance parameter, we extend the notion of *ν*. Let $\tau _j^l(x_{-l}) = E(\tau _j(X)\mid X_{-l} = x_{-l})$ and $E^l(x_{-l}) = E(X_l\mid X_{-l} = x_{-l})$, and define $\nu _l^1 = E^l$ and $\nu _l^2 = (\varLambda _1, \varLambda _2, \varLambda _c, \pi , \tau _j^l, E^l)$. We define the uncentered EIF’s corresponding to the EIF’s ${\tilde{\psi }}_{\varGamma _j^l}$ and ${\tilde{\psi }}_{\chi _j^l}$ as

7 $$\begin{aligned} \phi _{\varGamma _j^l}(O; \nu _l^2)&= [\varphi _j(O;\nu ) - \tau _j^l(X_{-l})][X_l - E^l(X_{-l})] \end{aligned}$$

8 $$\begin{aligned} \phi _{\chi ^l}(O; \nu _l^1)&= [X_l - E^l(X_{-l})]^2. \end{aligned}$$

The construction of the estimators ${\hat{\varGamma }}_j^{l,CF}$ and ${\hat{\chi }}^{l,CF}$ for $\varGamma _j^l$ and $\chi ^l$, respectively, now follow similar to the procedure in the ATE setting, as described in Appendix A. We note that estimation of $\chi ^l$ is given in Ziersen and Martinussen (2025), but is included here as well for completeness.

Since the estimators ${\hat{\varGamma }}_j^{l,CF}$ and ${\hat{\chi }}^{l,CF}$ depend on the extended nuisance estimators, we have to make additional assumption in order to derive the desired asymptotic linearity. Accordingly, we have the following result:

#### Theorem 2

For a fixed *l* and *j*, assume that for each fold *k=1,… , K* it holds that

(i) $\left( X_l - {\hat{E}}^l \right) ^2 \le M, \ a.s$ for all *n* and some *M>0.*
(ii) $$\left\Vert {\hat{\tau }}_j^l - \tau _j^l\right\Vert = o_p(n^{-1/4}).$$
(iii) $$\left\Vert {\hat{E}}^l - E^l\right\Vert = o_p(n^{-1/4})$$.

Then, if assumption B holds for each *k*, it follows that ${\hat{\varOmega }}_j^{l,CF} = \frac{{\hat{\varGamma }}_j^{l, CF}}{{\hat{\chi }}_j^{l, CF}}$ is asymptotically linear with influence function given by ${\tilde{\psi }}_{\varOmega _j^l}$ and hence

$$\sqrt{n}({\hat{\varOmega }}_j^{l,CF} - \varOmega _j^{l}) \overset{d}{\rightarrow }\ {\mathcal {N}}(0, P{\tilde{\psi }}_{\varOmega _j^l}^2).$$

#### Proof

See Appendix D. *□*

Assumptions *(i)-(iii)* in Theorem 2 refer to the nuisance estimators related to the conditional distribution of $X_l$ given $X_{-l}$. Regarding assumption B2 for ATE estimation, we discussed the double robustness properties of the cross-fitted estimator in relation to the convergence rates of the nuisance estimators, and we can add to that discussion the rates given in (*ii*) and (*iii*). We see that the estimator for our target parameter achieves parametric rates (asymptotic linearity) if the nuisance estimators related to $X_l|X_{-l}$ are estimated at $n^{-1/4}$-rate, adding to the notion of “double robustness”. The rate in assumption (*iii*) is known for many estimators, as it is an assumption on a typical regression estimator. Whether it is fulfilled depends on the type of the estimator used and possibly on the dimension *d* in relation to *n*, but we note that the assumption is to be considered rather mild, allowing for many types of data-adaptive estimators (see e.g. the discussion in Kennedy 2022, Sect. 4.3). For estimation of ${\hat{\tau }}_j^l$, we regress the CATE estimates $\left( {\hat{\tau }}_j(X_i)\right) _{i=1}^n$ onto $X_{-l} = \left( X_{i,-l}\right) _{i=1}^n$ in line with the approach suggested in Hines and Diaz-Ordaz (n.d.), and Ziersen and Martinussen (2025). This approach constitutes a certain type of meta-learning and convergence rates related to (*ii*) are generally less known compared to the regression in assumption (*iii*). We refer to Hines and Diaz-Ordaz (2023) for a discussion of a specific meta-learner termed the DR-learner (Kennedy 2023) for estimation of ${\hat{\tau }}_j^l$ (their analogy is termed ${\hat{\tau }}_s$) and convergence rates analogous to (*ii*).

Because of scale sensitivity, the estimate of $\varOmega _j^l$ may be of less interest than testing the null-hypothesis $H_0:\varOmega _j^l = 0$. A test statistic for $H_0$ can be defined by

$$\text{ TST}_j^l\equiv \frac{{\hat{\varOmega }}_j^{l,CF}}{\sqrt{{\hat{\sigma }}_{\varOmega _j^l}^{2,CF}/n}},$$

where the construction of ${\hat{\sigma }}_{\varOmega _j^l}^{2,CF}$ is given in Appendix A. The following corollary (analogous to Corollary 4 in Ziersen and Martinussen (2025)) gives the desired asymptotic properties of our test-statistic:

#### Corollary 1

Under the same setup as in Theorem 2, we have under the null-hypothesis, $H_0:\varOmega _j^l=0$, that

$$\text{ TST}_j^l\overset{D}{\longrightarrow }\ {\mathcal {N}}(0,1).$$

#### Proof

Since the variance estimator ${\hat{\sigma }}_{\varOmega _j^l}^{2,CF}$ is consistent (Ziersen and Martinussen 2025, Lemma 1), Theorem 2 together with Slutsky’s theorem and an application of the delta method gives the result. *□*

## Simulation study

We conduct simulation studies to investigate the proposed asymptotic properties of the estimators ${\hat{\psi }}_j^{CF}$ and ${\hat{\varOmega }}_j^{l,CF}$ under two different nuisance estimator settings with and without cross-fitting (i.e. setting *K=1*). In the following we set ${\hat{S}}(t\mid a,x) = \exp (-{\hat{\varLambda }}_1(t\mid a, x ) - {\hat{\varLambda }}_2(t\mid a, x ))$, ${\hat{S}}_C(t\mid a,x) = \exp (-{\hat{\varLambda_C }}(t\mid a, x))$ and ${\hat{F}}_j(t\mid a,x) = \int _0^t {\hat{S}}(u\mid a,x) \textrm{d}{\hat{\varLambda }}_j(u\mid a,x)$ for a given nuisance estimator ${\hat{\nu }} = ({\hat{\varLambda }}_C, {\hat{\varLambda }}_1, {\hat{\varLambda }}_2, {\hat{\pi }})$. For all cross-fitted estimators, we set *K=10*. In one nuisance estimator setting we consider correctly specified (semi)parametric nuisance estimators and in the other we use completely nonparametric estimators via random forest. The parametric nuisance estimators adhere to assumption B and so we would expect the target parameter estimators to perform according to theory both with and without cross-fitting. In case of nonparametric estimators, random survival forest are shown to adhere to assumption B3 in Cui et al. (2022), but it is unclear to what extend they admit rates corresponding to B2. Furthermore, the nonparametric estimators do not in general belong to a Donsker class, and we therefore expect the cross-fitted estimators to perform more in line with the theory compared to the non-cross-fitted version (see Chernozhukov et al. (2018) and Kennedy (2022) for a discussion on cross-fitted one-step estimators).

We consider data generated from the following models:

- $$X_l \sim \text {Unif}[-1,1], \ l = 1, \ldots , 4$$
- $$\pi (1\mid X) = \text {expit}(0.5X_1 + 0.5X_2)$$
- $$\lambda _1(t\mid A, X) = 0.0025\cdot 2t^{2} \exp (-X_1 - X_2 - 0.2X_3 + A(0.5X_1 - 0.3X_2 - 2))$$
- $$\lambda _2(t\mid A, X) = 0.00025\cdot 2t^{2} \exp (-X_1 - X_2 - 0.2X_3 + A)$$
- $$\lambda _c(t\mid A, X) = 0.00025\cdot 2t^{2} \exp (-0.5X_1)$$.

Note that the above hazard functions correspond to Cox models with baseline hazards given by a Weibull hazard, $bkt^{k-1}$, where *b* and *k* are the scale and shape parameter, respectively. We consider four sample size settings of *n=250, 500, 750, 1000*, and for each setting we run 1000 simulations. For each simulation we generate data according to the models above and estimate the target parameters according to specifications given below.

Additionally, we conducted the same simulation study as described above but with correlated covariates following a multivariate normal distribution. The results are similar to the uniform setting presented here, and they can be found in Appendix E.

### Average treatment effect

For estimation of ${\hat{\psi }}_1^{CF}$ we choose the time-horsizon $t^*=30.$ Under the model, the folliwing true values were approximated by Monte-Carlo simulation using $10^8$ draws from the true distribution, $\psi _1(P_0) \approx -9.62$, $\psi _2(P_0) \approx 4.06$, $E\{L_1(0,t^*|1, X)\} \approx 2.63$, $E\{L_1(0,t^*|0, X)\} \approx 12.25$, $E\{L_2(0,t^*|1, X)\} \approx 5.28$, $E\{L_2(0,t^*|0, X)\} \approx 1.22$. We consider two nuisance settings for estimation of ${\hat{\nu }}$ (here dropping *k* from the notation). In one setting ${\hat{\nu }}$ consists of correctly specified Cox models with corresponding Breslow estimators for the cumulative hazards and a correctly specified logistic regression for the propensity score model. This setting is abbreviated **cor** in Table 1. In the second setting we estimate the cumulative hazards by random survival forests (Ishwaran et al. 2008) as implemented in the R-package randomForestSRC with default tuning parameters (see Ishwaran and Kogalur 2023 for documentation), and we estimate the propensity score by random forest, again with the implementation and tuning parameters given by randomForestSRC. This setting is abbreviated **RF**. Furthermore, each setting will be given the suffix **CF** if cross-fitting is used.

Table 1 gives the results for ATE-estimation. For correctly specified nuisance estimators the bias decreases with *n* and standard deviations decrease at an approximately $n^{-1/2}$-rate. With a coverage around 0.95, even for relatively small *n*, it looks as if the estimator follows the asymptotic distribution from Theorem 1. Surprisingly though, cross-fitting seems to decrease the bias of the estimator with correctly specified nuisance parameters even further. Overall we find that the estimator performs as expected for correctly specified (semi)parametric nuisance estimators.

**Table 1:** Results of 1000 simulations of estimators of $\psi _1$ with varying nuisance estimators, with and without cross-fitting, for sample sizes *n=250, 500, 750, 1000.*

n	method	bias	SD	mean SE	coverage
250	**cor**	−0.1521	0.9424	0.9746	0.9550
	**corCF**	−0.0494	0.9701	1.0340	0.9620
	**RF**	−0.3501	0.9020	0.5644	0.7520
	**RFCF**	0.0953	1.2012	1.3429	0.9710
500	**cor**	−0.0980	0.6946	0.6893	0.9570
	**corCF**	−0.0538	0.7040	0.7084	0.9550
	**RF**	−0.3576	0.6750	0.3957	0.6800
	**RFCF**	−0.0383	0.7912	0.8799	0.9680
750	**cor**	−0.0665	0.5650	0.5633	0.9480
	**corCF**	−0.0377	0.5684	0.5735	0.9500
	**RF**	−0.3269	0.5538	0.3237	0.6750
	**RFCF**	−0.0678	0.6394	0.6970	0.9670
1000	**cor**	−0.0265	0.4724	0.4884	0.9540
	**corCF**	−0.0046	0.4754	0.4949	0.9570
	**RF**	−0.2800	0.4636	0.2808	0.6950
	**RFCF**	−0.0319	0.5383	0.5943	0.9710

The abbreveations of the methods are read as follows: **cor** corresponds to the nuisance parameters $\varLambda _1, \varLambda _2, \varLambda _c, \pi$ estimated by correctly specified Cox and logistic regressions, and **RF** corresponds to the same parameters estimated by Random Forest. A suffix **CF** indicates that cross-fitting was employed in estimation of $\psi _j$. The tables gives the bias, empirical standard deviation (SD), mean of the estimated standard error (mean SE), and coverage.

For the nuisance estimators using random forests, we see a non-vanishing bias for the non-cross-fitted estimators. Furthermore, the standard errors are underestimated compared to the empirical standard deviation of the estimators which, together with the bias result in undercoverage. When using cross-fitting together with random forests, the bias disappears on roughly the same order as the correctly specified parametric estimators (without cross-fitting), and the standard deviation of the estimator seem to be on roughly $\sqrt{n}$-rate, as with the correctly specified nuisance parameters. The standard errors seem to be slighty overestimated, though, resulting in a slight overcoverage. This might be due to the hyperparameter choices for the random forests or to inherent limitations on the convergence rates of random forests in regards to the number of covariates (Biau 2012).

### Best partially linear projection

For estimation of ${\hat{\varOmega }}_1^{l,CF}$, we set $t^*=30$. The true value of the target parameters are approximately $(\varOmega _1^1, \varOmega _1^2, \varOmega _1^3, \varOmega _1^4) = (4.949, 3.137, 0.737, 0)$. We consider two nuisance settings for estimation of ${\hat{\nu }}^2$ (here dropping *k* from the notation). In one setting ${\hat{\nu }}^2$ consists of correctly specified Cox models with corresponding Breslow estimators for the cumulative hazards, and a correctly specified logistic regression for the propensity score model, and ${\hat{\tau }}^l$ and ${\hat{E}}^l$ are estimated with a generalised additive model (GAM) including spline smoothing of each term but without interactions as implemented in the R-package mgcv. This setting will be abbreviated **cor** in figures going forward. In the second, all nuisance parameters are estimated by random (survival) forests with default tuning parameters as implemented in the R-package randomForestSRC. This setting will be abbreviated **RF**. Furthermore, each setting will be given the suffix **CF** if cross-fitting is used.

**Fig. 1:**
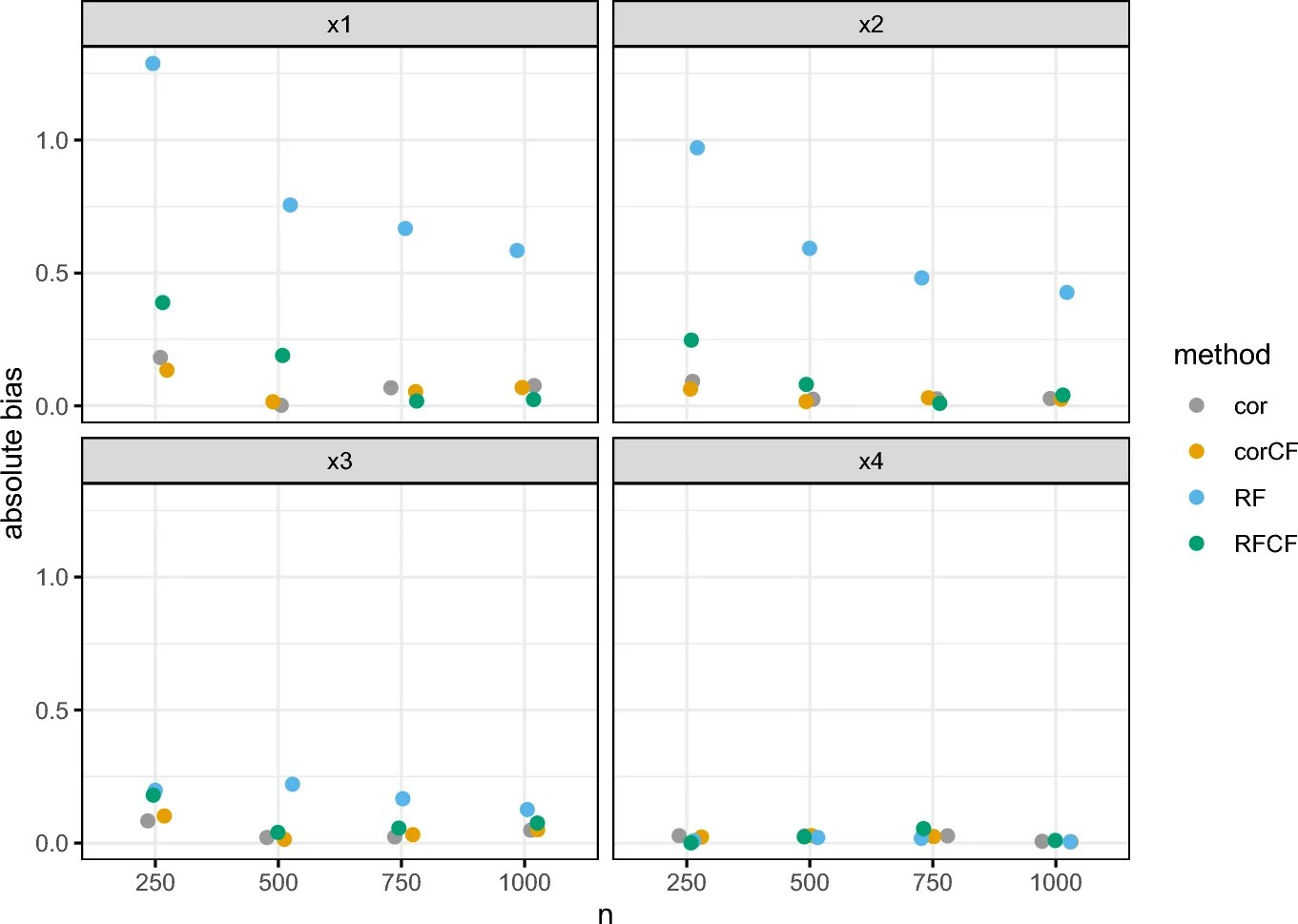
Results based on 1000 simulations of the estimators of $\varOmega _1^l$ with for *l=1,… ,4* with varying nuisance estimators and across sample sizes *n=250, 500, 750, 1000*. The plot shows the absolute bias of the estimators, where **cor** corresponds to the nuisance parameters $\varLambda _1, \varLambda _2, \varLambda _c, \pi$ estimated by correctly specified Cox and logistic regressions, and **RF** corresponds to the same parameters estimated by Random Forest. A suffix **CF** indicates that cross-fitting was employed. The true values are $(\varOmega _1^1, \varOmega _1^2, \varOmega _1^3, \varOmega _1^4) = (4.949, 3.137, 0.737, 0)$.

**Fig. 2:**
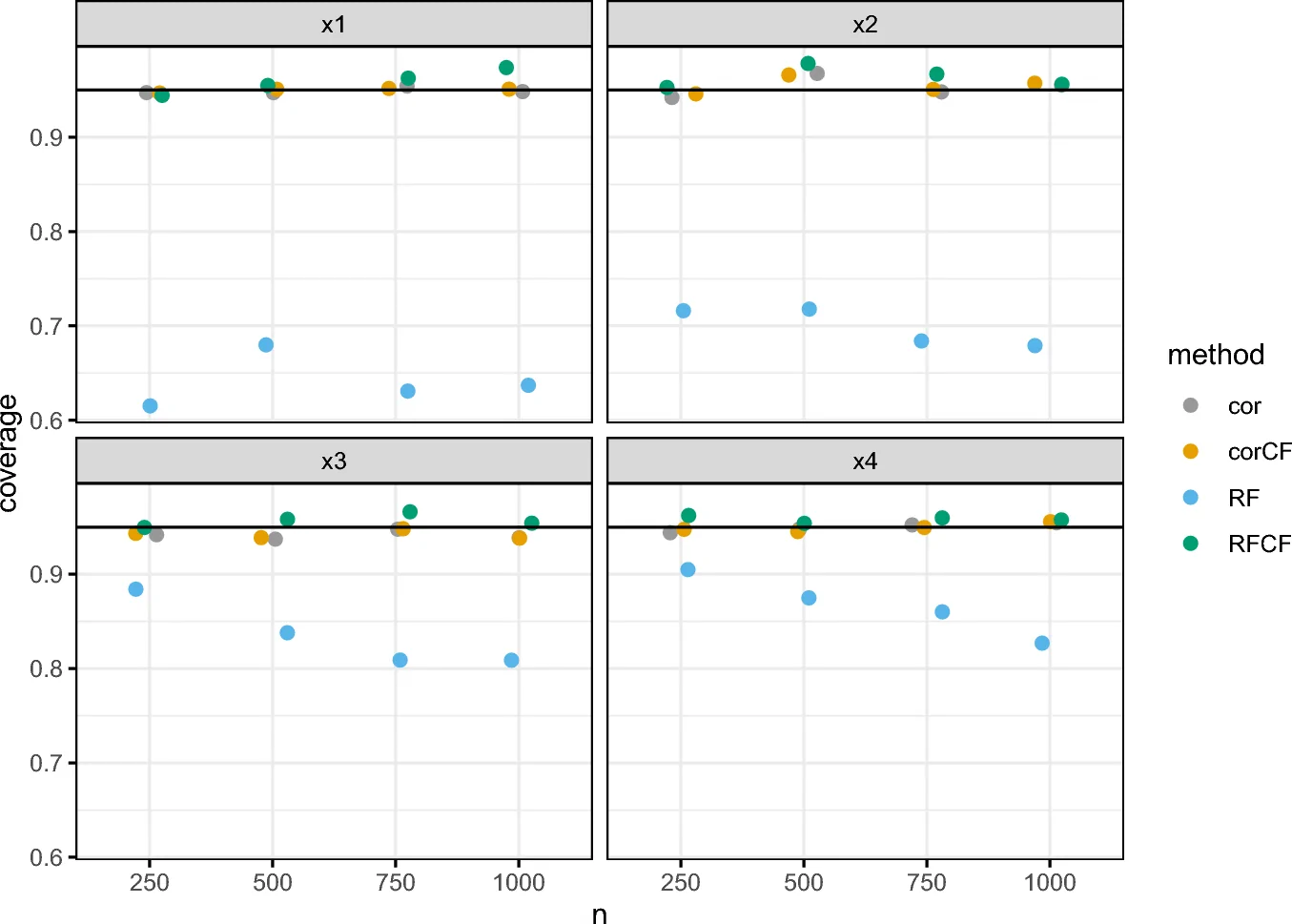
Results based on 1000 simulations of the estimators of $\varOmega _1^l$ with for *l=1,… ,4* with varying nuisance estimators and across sample sizes *n=250, 500, 750, 1000*. The plot shows coverage of the estimators, where **cor** corresponds to the nuisance parameters $\varLambda _1, \varLambda _2, \varLambda _c, \pi$ estimated by correctly specified Cox and logistic regressions, and **RF** corresponds to the same parameters estimated by Random Forest. A suffix **CF** indicates that cross-fitting was employed. The black line indicates a coverage of 0.95.

**Fig. 3:**
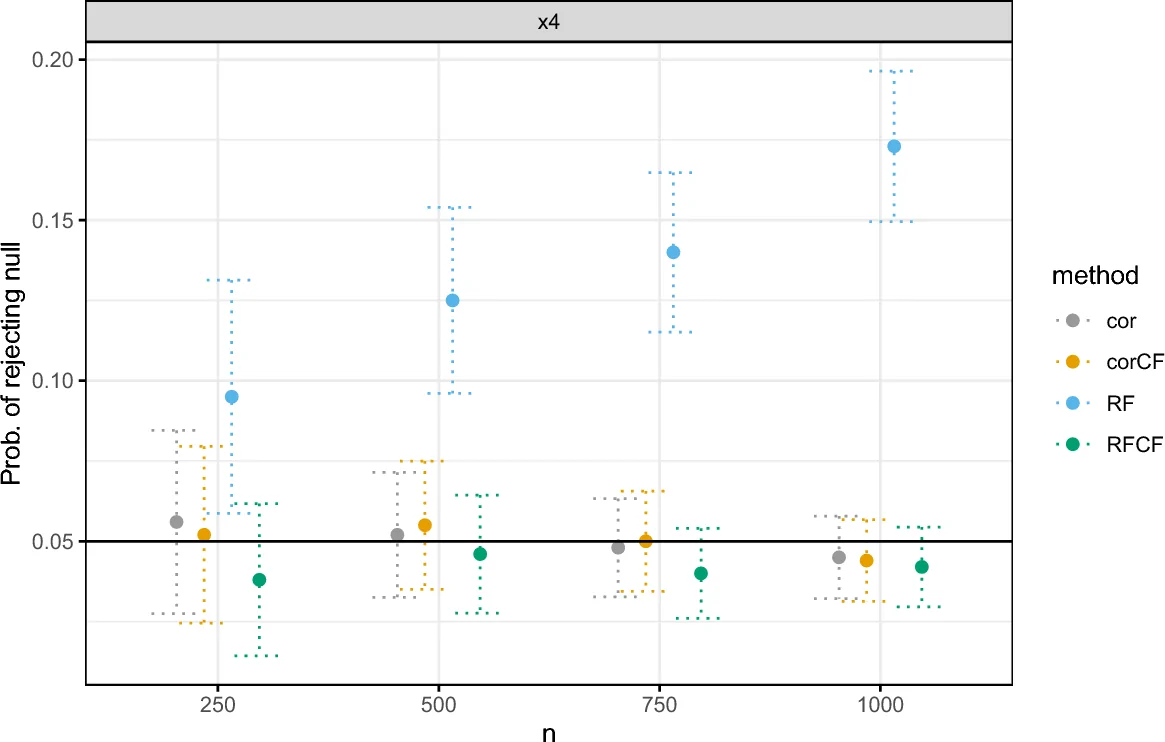
Results based on 1000 simulations of the test statistic corresponding to the test $H_0:\varOmega _1^4 = 0$ with varying nuisance estimators and across sample sizes *n=250, 500, 750, 1000*. The plot shows probability of rejecting $H_0$, which equals the type-1 error as $\varOmega _1^4=0$. **cor** corresponds to the nuisance parameters $\varLambda _1, \varLambda _2, \varLambda _c, \pi$ estimated by correctly specified Cox and logistic regressions, and **RF** corresponds to the same parameters estimated by Random Forest. A suffix **CF** indicates that cross-fitting was employed.

In Fig. 1, we see the absolute bias for estimation of ${\hat{\varOmega }}_1^l$, *l = 1, … , 4*, for the different nuisance settings. In general, we see that **cor** and **corCF** perform similarly across all sample sizes and across all *l*, with a bias converging to zero. The **RFCF**-setting performs slightly worse than **cor** and **corCF** for small *n*, but has approximately similar performance for large *n*, whereas **RF** has a large bias for large enough values of $\varOmega _1^l$. Generally, the estimators seem to perform as we would expect according to Theorem 2 in terms of bias. The coverage of the estimators are presented in Fig. 2. The settings **cor**, **corCF** and **RFCF** all exhibit approximately nominal coverage across *n* and *l*, whereas **RF** has poor coverage. Again, this is in line with our expectations. Figure 3 gives the estimated probability of rejecting $H: \varOmega _1^4 = 0$, i.e. the type-1 error (since $\varOmega _1^4 = 0$ in our data generating mechanism), together with Monte Carlo confidence intervals. The type-1 error is approximately 0.05 for all *n*, except for **RF** where the type 1-error increases with *n*.

**Fig. 4:**
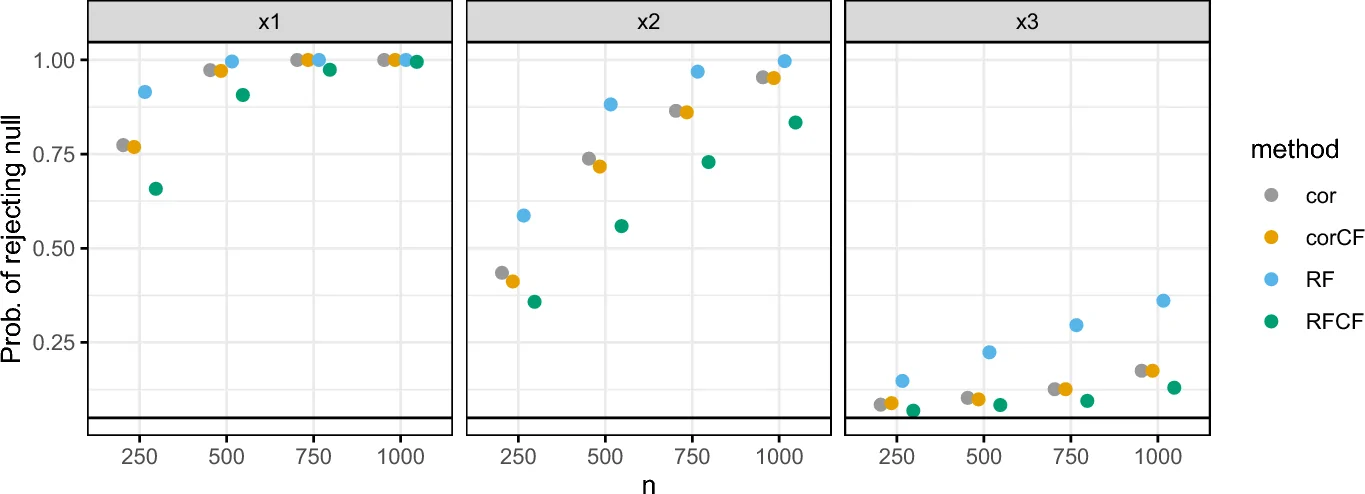
Results based on 1000 simulations of the test statistic corresponding to the test $H_0:\varOmega _1^l = 0$, for *l=1,2,3*, with varying nuisance estimators and across sample sizes *n=250, 500, 750, 1000*. The plot shows probability of rejecting $H_0$, which corresponds to the power of the test as $\varOmega _1^l> 0$ for *l=1,2,3*. **cor** corresponds to the nuisance parameters $\varLambda _1, \varLambda _2, \varLambda _c, \pi$ estimated by correctly specified Cox and logistic regressions, and **RF** corresponds to the same parameters estimated by Random Forest. A suffix **CF** indicates that cross-fitting was employed.

Lastly, Fig. 4 shows the probability of rejecting $H: \varOmega _1^l = 0$, *l = 1,2,3*, which correspond the power of the test. Interestingly, using data-adaptive estimation of the nuisance parameters seem to decrease the power of the test $TST_1^l$.

## Application

To demonstrate the methods outlined in the previous sections, we consider the study by Kessing et al. (2024). In this study, the potential non-responses to 17 different antidepressants are compared based on data from the Danish national registers. Patients enter the study at their first diagnosis with major depressive disorder from a psychiatric hospital. Their treatment, in terms of a specific antidepressant, is defined as the first purchase of an antidepressant after discharge from the hospital, which also determines the index date. The main outcome is time to non-response, defined as a switch to or add-on of another antidepressant or antipsychotic medicine or readmission to a psychiatric hospital with a major depressive disorder. Competing risk for the time to non-response was admission to a psychiatric hospital with a higher order psychiatric diagnosis (bipolar disorder, schizophrenia or organic mental disorder) or death. The 17 antidepressants are categorised into six groups (SSRI, NARI, SNRI, NaSSA, TCA, and others) and within each group a reference drug is chosen to which the other drugs in that group are compared. The estimand for each comparison is the average treatment effect on the risk of non-response within two years after index date, i.e., it is defined as ${{\,\mathrm{\textrm{E}}\,}}\{F_1(t\mid A=1, X) - F_1(t\mid A=0, X)\}$, where $F_1$ is the conditional cumulative incidence function for non-response, *A=0* denotes the reference drug, *A=1* is the comparitor and *t=2* corresponds to the time-horizon. The study includes patients from 1995-2018 and not all of the antidepressants considered were available on the marked in the entire period. Accordingly, for each comparison, a minimum date is set for which both drugs in comparison were available and all patients with index dates prior to the minimum date are excluded. Additionally, censoring was defined at emigration or end of follow-up.

**Table 2:** Confounders in Kessing et al. (2024)

Annotation	Explanation
age	age in years at index-date
sex	female, male
Secondary diagnosis from the psychiatric hospital at inclusion. The annotations reflect the ICD-10 codes used in the definition:	
F10-19	other psychiatric disorders
F40-48	neurotic, stress-related and somatoform disorders
F60-69	personalitiy disorders
Diagnosis with somatic disorder within 10 years prior to index date. The annotations are given in form of the corresponding ICD-10 chapter:	
I	infections
II	neoplasms
III	blood diseases
IV+IX+X	endocrine, nutritional, and metabolic diseases and diseases of the circulatory or respiratory system
VI-VIII	diseases of the nervous system, eye and ear
XI	diseases of the digestive system
XII	diseases of the skin and subcutaneous tissue
XIII	diseases if the musculoskeletal system
XIX	physical lesions and poisoning)

For the sake of illustration we restrict ourselves to the comparison of Setraline (reference drug, *n=14416*) and Escitalopram (comparitor, *n=7508*). The censoring and event rates for non-response and competing risks, respectively, at time $t^*=730.5$ days were 0.135, 0.433 and 0.066. Kessing et al. (2024) used the G-formula with $F_1$ estimated by cause-specific Cox regressions (Ozenne et al. 2020) to estimate the ATE. To control for confounding, the Cox-regressions were adjusted for the covariates in Table 2. Here, the secondary psychiatric diagnoses and the somatic disorders at baseline are indicator variables and they are defined as having a diagnosis within the specific groups, according to ICD10 codes, within 10 years prior to the index date. The ATE was estimated to be 0.10 (0.09, 0.12), that is, the probability of non-response was 0.1 higher amongst patients treated with Escitalopram within two years after treatment initiation. For comparison, instead of defining the treatment effect through the cumulative incidence function, $F_1$, we consider estimation of the ATE and the best partially linear projection based on the number of life-years-lost estimands defined in Sect. 3. That is, we consider estimation of $\psi _1$ and $\varOmega _1^l$ with $t^* = 730.5$ (2 years). We include the same confounders as in Kessing et al. (2024) with the exception that age is included as a numeric variable instead of a categorised version. The target parameters are estimated with the cross-fitted estimators described in Sect. 4 with all nuisance parameters estimated by random forests (as described in the simulation study) and *K=10* folds.

The ATE is estimated to 48.96 (40.02, 57.90). The interpretation here is that patients on Setraline on average lost 49 “healthy” days less before two years after treatment initiation due to non-response compared to patients who start on Escitalopram, where “healthy” is meant as time without a non-response event or a competing event. For comparison, we also estimated the effect of treatment on the competing event, i.e. $\psi _2$, to 4.06 (−0.54, 8.66). Table 3 shows the estimates ${\hat{\varOmega }}_1^{l,CF}$, for *l* given by each confounder in Table 2, together with a p-value associated with the test statistic $\text{ TST}_1^l$.

**Table 3:** Estimates of ${\hat{\varOmega }}_1^{l,CF}$ ranked according to the p-value associated with the test of $H:\varOmega _1^l = 0$, for *l* ranging over different covariates

	age	sex	XIII	XI	F60-69	II	VI-VIII	IV+IX+X	F40-48	III	XIX	XII	I	F10-19
${\hat{\varOmega }}_1^{l,CF}$	−0.85	18.7	−28.3	−16.7	15.1	17.4	8.88	4.38	5.21	−4.80	−2.85	−2.00	−0.41	0.23
p-value	0.03	0.04	0.17	0.27	0.30	0.36	0.40	0.73	0.76	0.82	0.83	0.88	0.98	0.98

The acronyms in the table are described in Table 2. Random Forest was used for all nuisance parameter estimators. The data comes from the study Kessing et al. (2024), and the outcome is time to non-response, which is defined as a switch in psychiatric treatment or re-hospitalization at psychiatric ward. The treatment effect was defined as the difference in the number of healthy days lost (days without switch of treatment or re-hospitalization) due to non-response before two years after treatment initiation between Escitalopram and Setraline.

The p-values indicate that potential treatment effect heterogeneity can be attributed to sex and age, while the treatment effect does not seem to vary across any of the other variables. Specifically, since $\varOmega _1^l$ is defined as the projection of the CATE function onto the partially linear model, the estimates related to sex and age can be interpreted as regression coefficients. The CATE function is defined as the difference in number of healthy days lost due to non-response between Escitalopram and Setraline, and the estimate ${\hat{\varOmega }}_1^{sex, CF}=18.69$ then corresponds to the treatment effect being larger among women compared to men, i.e., the difference in number of healthy days lost due to non-response between Escitalopram and Setraline was 19 days larger among women than men. This interpretation is of course relying on the partially linear model to hold for the CATE function, but as the parameter still measures the association between $\tau_1$ and sex, when the partially linear model does not hold, we would still conclude, that the treatment effect is larger among women.  

## Discussion

In this paper, we have introduced the causal effect of a treatment on the number of life-years lost due to a specific event. We have shown that the ATE and the CATE are identifiable from the observed data under common assumptions found throughout the causal inference and survival literature. Different measures of treatment effect in the presence of competing risk are available in causal inference (Rytgaard et al. 2023; Ozenne et al. 2020; Stensrud et al. 2021, 2022; Martinussen and Stensrud 2023) and the treatment effect studied in this paper adds a new interpretability compared to existing variants. One advantage is that the treatment effect is defined on the time scale of the study and thus provides a quantity that is easy to communicate to non-statisticians, whereas treatment effects based on the cumulative incidence function are harder to communicate. As is common when assessing the treatment effect in the presence of competing risk, the effect of a treatment on the number of life years lost due to a specific event depends on the effect of the treatment on both the hazard of the event of interest and on the competing event. As such, one can in principle conclude that there is an effect of treatment, even when all of the effect is driven by the effect on the competing event. Martinussen and Stensrud (2023) provide a measure of separable treatment effects based on the cumulative incidence function, which allow one to estimate the effect of treatment only driven by the intensity of the event of interest under additional causal assumptions. An interesting avenue for future research is to extend their method to the number of life years lost due to a specific event.

We have provided an estimator of the ATE based on semiparametric efficiency theory, which allows for data-adaptive estimation of the nuisance parameters. The estimator is efficient in the nonnparametric model with variance given by the efficient influence function. One of the assumptions needed to ensure the asymptotic results relies on convergence of a remainder term on $n^{-1/2}$-rate (assumption B2), which is reminiscent of a similar assumption in the causal inference literature with censored data (Westling et al. 2024; Rytgaard et al. 2023). Without assuming absolute continuity of the cumulative hazard estimators, it is difficult to obtain an equivalent double rate-robustness property as is seen in the literature on uncensored data (e.g. Kennedy 2022; Hines et al. 2022; Chernozhukov et al. 2018; van der Laan and Rose 2011). Accordingly, we conducted a simulation study, where the nuisance parameters were estimated by different variants of random forests, which confirmed that the estimator performed according to asymptotic results when using data-adaptive nuisance estimators.

Lastly, we extended a measure of treatment effect heterogeneity, termed the best partially linear projection (Ziersen and Martinussen 2025), to the CATE-function defined on the number of life-years lost due to a specific event. The measure asserts the importance of a given covariate on the treatment effect, but with competing risk the interpretation is more delicate compared to the survival setting. When the effect of treatment on the competing event is large, one can imagine scenarios where the importance of one covariate on the CATE is driven by the effect on the competing event, and an assertion of importance (shown in Sect. 6) based on the event of interest might be misleading. In such scenarios, one can switch the event of interest and competing event to get a full picture.

## Figures and Tables

**Fig. 5:**
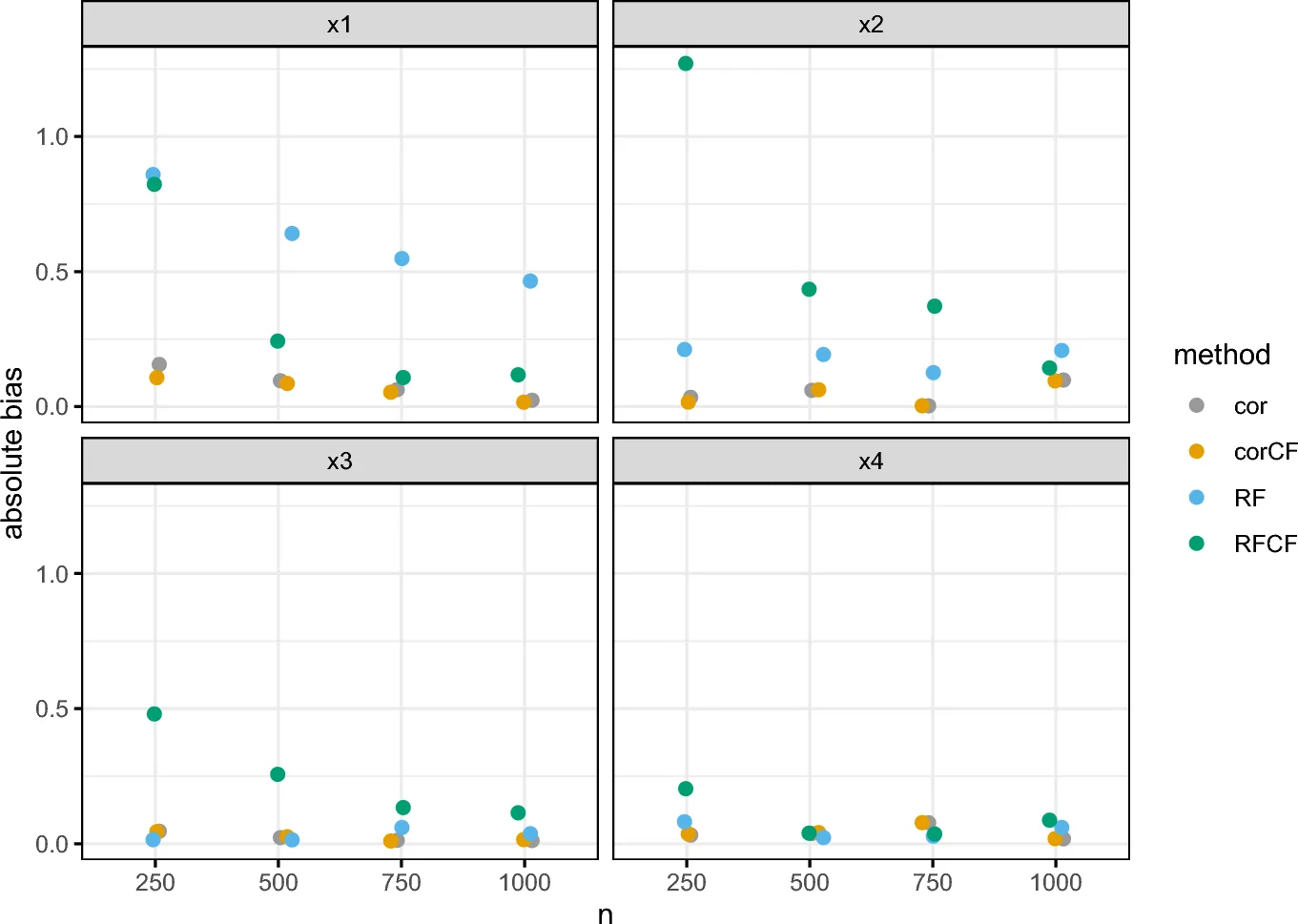
Results based on 1000 simulations of the estimators of $\varOmega _1^l$ with for *l=1,… ,4* with varying nuisance estimators and across sample sizes *n=250, 500, 750, 1000*. The covariates are correlated and follow a multivariate normal distribution. The plot shows the absolute bias of the estimators, where **cor** corresponds to the nuisance parameters $\varLambda _1, \varLambda _2, \varLambda _c, \pi$ estimated by correctly specified Cox and logistic regressions, and **RF** corresponds to the same parameters estimated by Random Forest. A suffix **CF** indicates that cross-fitting was employed. The true values are $(\varOmega _1^1, \varOmega _1^2, \varOmega _1^3, \varOmega _1^4) = (4.507, 2.640, 0.664, 0.008)$.

**Fig. 6:**
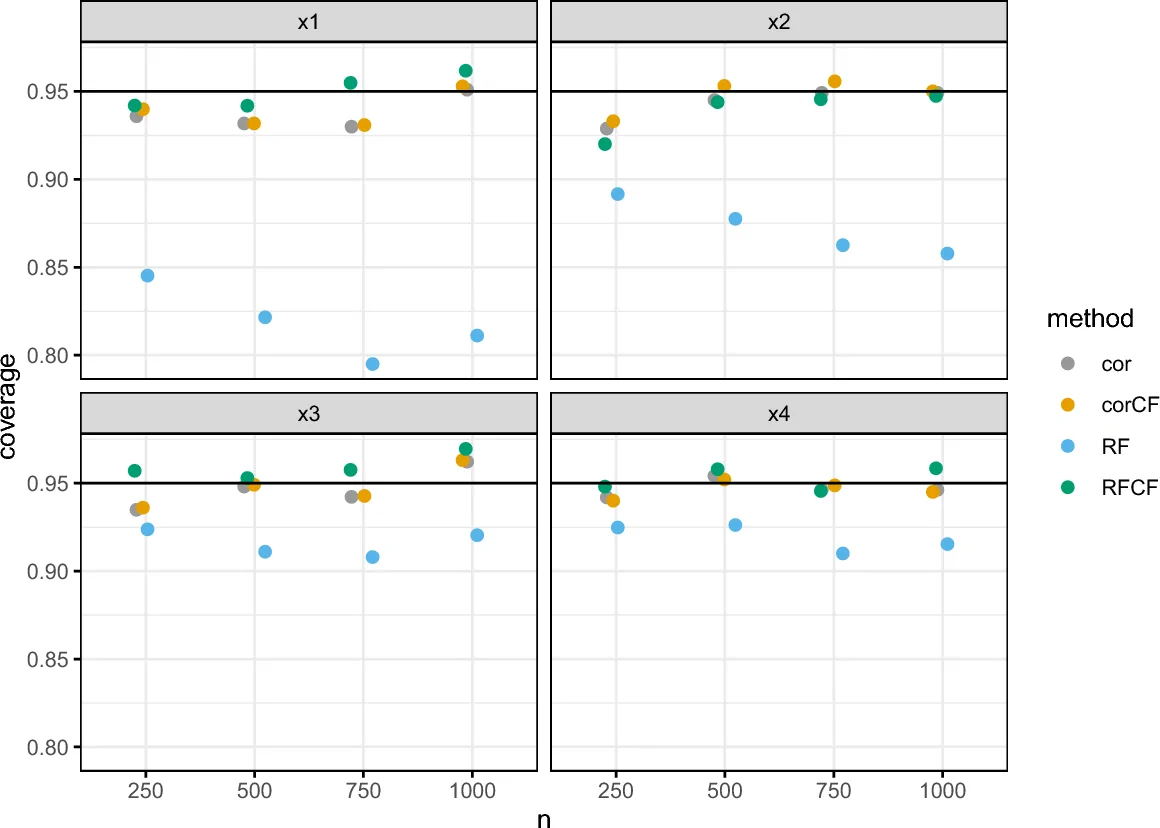
Results based on 1000 simulations of the estimators of $\varOmega _1^l$ with for *l=1,… ,4* with varying nuisance estimators and across sample sizes *n=250, 500, 750, 1000*. The covariates are correlated and follow a multivariate normal distribution. The plot shows coverage of the estimators, where **cor** corresponds to the nuisance parameters $\varLambda _1, \varLambda _2, \varLambda _c, \pi$ estimated by correctly specified Cox and logistic regressions, and **RF** corresponds to the same parameters estimated by Random Forest. A suffix **CF** indicates that cross-fitting was employed. The black line indicates a coverage of 0.95

**Fig. 7:**
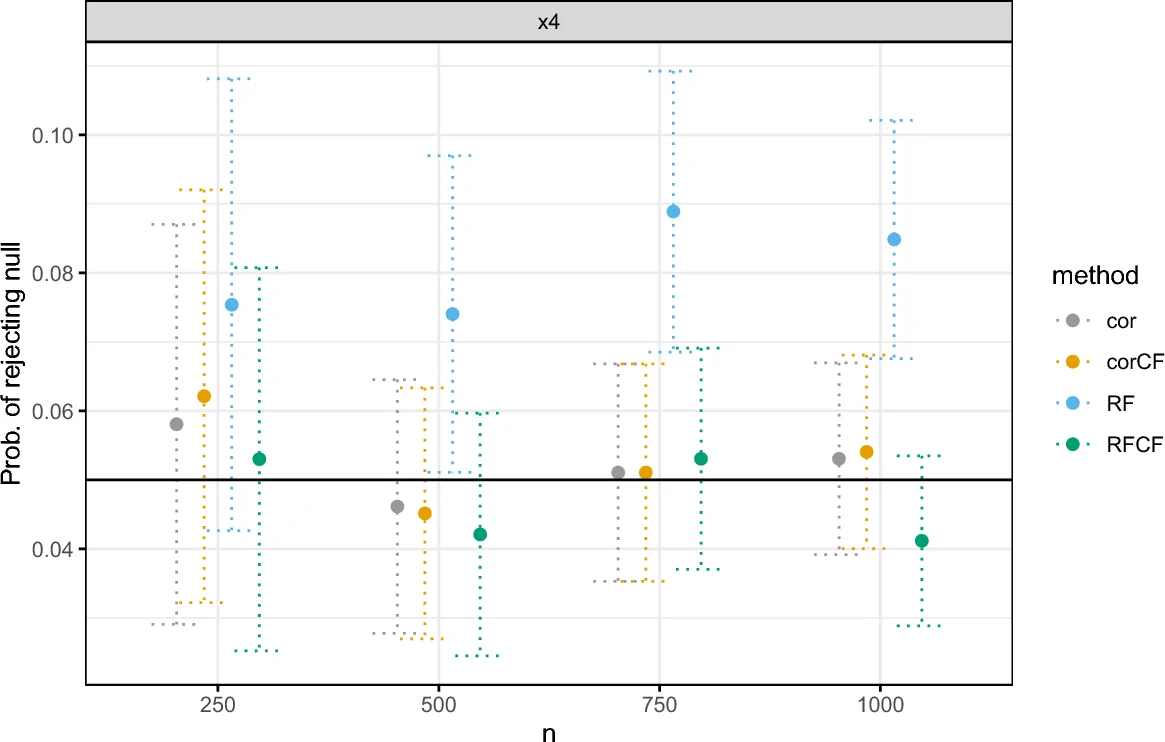
Results based on 1000 simulations of the test statistic corresponding to the test $H_0:\varOmega _1^4 = 0$ with varying nuisance estimators and across sample sizes *n=250, 500, 750, 1000*. The covariates are correlated and follow a multivariate normal distribution. The plot shows probability of rejecting $H_0$, with the true $\varOmega _1^4=0.008$. **cor** corresponds to the nuisance parameters $\varLambda _1, \varLambda _2, \varLambda _c, \pi$ estimated by correctly specified Cox and logistic regressions, and **RF** corresponds to the same parameters estimated by Random Forest. A suffix **CF** indicates that cross-fitting was employed

**Fig. 8:**
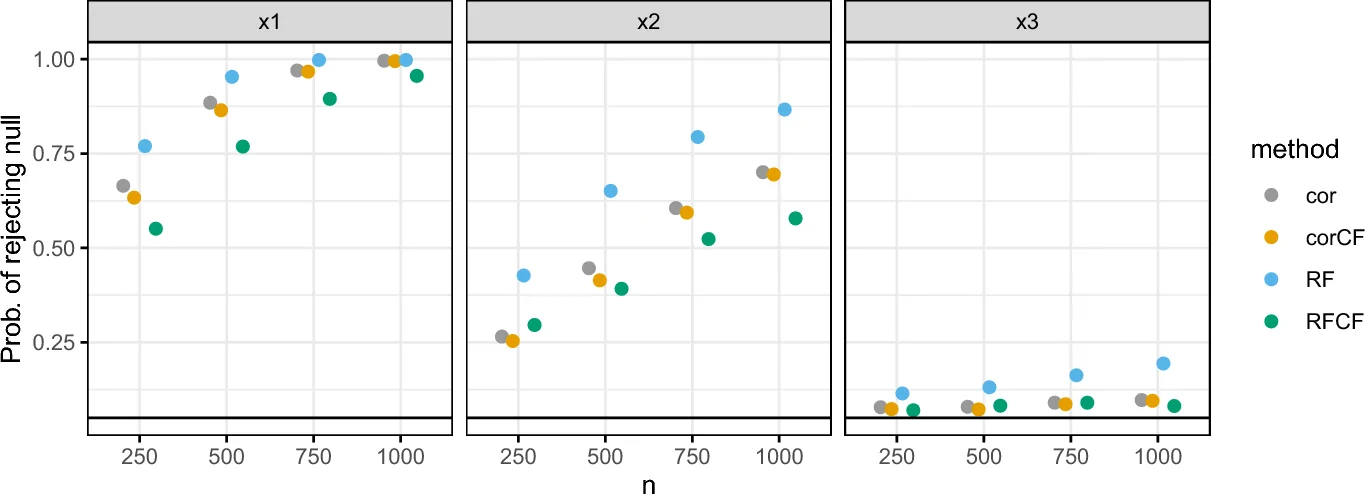
Results based on 1000 simulations of the test statistic corresponding to the test $H_0:\varOmega _1^l = 0$, for *l=1,2,3*, with varying nuisance estimators and across sample sizes *n=250, 500, 750, 1000*. The covariates are correlated and follow a multivariate normal distribution. The plot shows probability of rejecting $H_0$, which corresponds to the power of the test as $\varOmega _1^l> 0$ for *l=1,2,3*. **cor** corresponds to the nuisance parameters $\varLambda _1, \varLambda _2, \varLambda _c, \pi$ estimated by correctly specified Cox and logistic regressions, and **RF** corresponds to the same parameters estimated by Random Forest. A suffix **CF** indicates that cross-fitting was employed

## Data Availability

Implementation of the methods as well as code for reproducing the simulation study is found at https://github.com/simonziersen/causal-effect-on-the-number-of-life-years-lost. Data used in the application are comprised of patient sensitive information and are not publicly available.

## Appendix A On nonparametric estimation and efficiency theory

We give a brief introduction to semiparametric efficiency theory (Bickel et al. 1993; Van Der Vaart 2000, Ch. 25; van der Laan and Rose 2011) used to construct and analyze the estimators from Sect. 4. For a nice introduction, with the aim of leveraging the theory to construct nonparametric estimators incorporating data-adaptive methods, see Kennedy (2022).

For a general target parameter *ψ*, an estimator ${\hat{\psi }}$ is said to be asymptotically linear if it can be written on the form ${\hat{\psi }} - \psi = {\mathbb {P}}_n\mathbb{I}\mathbb{F} + o_p(n^{-1/2})$ with $P\mathbb{I}\mathbb{F}=0$. The function $\mathbb{I}\mathbb{F}$ is called the influence function of the estimator ${\hat{\psi }}$, and it characterizes the asymptotic distribution of the estimator. This can be seen by applying the central limit theorem together with Slutsky’s lemma, from which $\sqrt{n}({\hat{\psi }}-\psi ) \overset{D}{\rightarrow } {\mathcal {N}}(0, P\mathbb{I}\mathbb{F}^2)$. If the target parameter is differentiable at *P* as a map $\psi :{\mathcal {M}}\rightarrow {\mathbb {R}}$, there exist a unique function, say ${\tilde{\psi }}$, associated to the pathwise derivative of the parameter, which characterizes the information bound of any regular estimator. The function ${\tilde{\psi }}$ is called the efficient influence function (EIF), and any estimator is regular and asymptotically efficient, if it is asymptotically linear with influence function given by the EIF (Van Der Vaart 2000, Ch. 25.3).

Since the EIF is uniquely determined by the target parameter and the model, it can be calculated without reference to any estimator. Once it is known, several techniques exist for constructing estimators that are asymptotically linear with the EIF as their influence function (van der Laan and Rose 2011; Chernozhukov et al. 2018; Kennedy 2022; Hines et al. 2022). We focus on the so-called *one-step estimator*, which is defined as

$${\hat{\psi }}^{OS} = \psi ({\hat{P}}) + {\mathbb {P}}_n{\tilde{\psi }}(\cdot ;{\hat{P}}),$$

where ${\hat{P}}$ is obtained from some (possibly) data-adaptive estimators. When the EIF is linear in the target parameter, i.e., ${\tilde{\psi }} = \varphi - \psi$, for some function *φ (O;P)*, the one-step estimator becomes

$$\begin{aligned} {\hat{\psi }} = {\mathbb {P}}_n \varphi (\cdot ;{\hat{P}}). \end{aligned}$$

In the rest of this section we assume this form of the one-step estimator, since all estimators in the main text of the article take this form.

In order to show that the one-step estimator is asymptotically linear, one typically relies on a certain decomposition involving an empirical process term and a remainder term, which are both required to be $o_p(n^{-1/2})$. The former can be obtained if ${\hat{P}}$ is assumed to belong to a Donsker class, but this requirement has been shown to be too restrictive for some data-adaptive estimators, and a certain type of sample splitting, termed *cross-fitting*, has to be applied to the one-step estimator in order to obtain the required convergence rate (Chernozhukov et al. 2018; Kennedy 2022).

To define the sample splitting involved in constructing the cross-fitted estimator, let ${\boldsymbol{i}} = (i_1, i_2, \ldots , i_n)$ be an index vector drawn from an *n*-dimensional multinomial distribution with *K* events with probability $p_k=\frac{1}{K}$, *k=1,… , K*, for the *k*’th event. Define the index sets ${\mathcal {T}}_k = \{j : i_j = k\}$ for *k=1… K* such that $\{1, \ldots , n\} = {\dot{\cup }}_{k=1}^K {\mathcal {T}}_k$, where ${\dot{\cup }}$ denotes the disjoint union. Corresponding to the index sets, we define *K* disjoint data splits by ${\mathcal {V}}_k = \{O_j: i_j = k\}$ such that ${\mathcal {O}} = {\dot{\cup }}_{k=1}^K {\mathcal {V}}_k$, and we define *K* leave-out data splits by ${\mathcal {V}}_{-k} = {\dot{\cup }}_{j \ne k} {\mathcal {V}}_j$.

To construct the cross-fitted one-step estimator corresponding to *ψ*, let ${\hat{P}}_{-k}$ be an estimate of *P* based on data in the *k*’th leave out sample, ${\mathcal {V}}_{-k}$, and let ${\mathbb {P}}_n^k$ be the empirical measure of $O \in {\mathcal {V}}_k$. We define the K-fold cross-fitted estimator of *ψ* as

9 $$\begin{aligned} {\hat{\psi }}^{CF} = \sum _{k=1}^K \frac{n_k}{n} {\mathbb {P}}_n^k \varphi (\cdot ; {\hat{P}}_{-k}) = \frac{1}{n} \sum _{k=1}^K \sum _{i \in {\mathcal {T}}_k} \varphi (O_i;{\hat{P}}_{-k}), \end{aligned}$$

where $n_k$ is the number of observations in the *k*’th data split. The construction of the cross-fitted estimator detailed here is quite general, see Kennedy (2022) for a nice discussion and comparison to the one-step estimator without cross-fitting.

As mentioned above, the cross-fitted one-step estimator will be asymptotically linear if the associated empirical process term and remainder term converge in probability on $n^{-1/2}$-rate. Then $\sqrt{n}({\hat{\psi }}^{CF} - \psi ) \overset{D}{\longrightarrow } {\mathcal {N}}(0, P{\tilde{\psi }}^2)$, and the variance of the EIF can be estimated consistently, under the same assumptions required for asymptotic linearity of ${\hat{\psi }}^{CF}$ (Ziersen and Martinussen 2025, Lemma 1), by the cross-fitted plug-in estimator

$${\hat{\sigma }}_{\psi }^{2,CF} = \sum _{k=1}^K \frac{n_k}{n} {\mathbb {P}}_n^k\left( \varphi (\cdot ; {\hat{P}}_{-k}) - {\hat{\psi }}^{CF}\right) ^2,$$

and the standard error of ${\hat{\psi }}^{CF}$ is given by $\sqrt{{\hat{\sigma }}_{\psi }^{2,CF}/n}$.

The estimators in the main text follow this exact recipe, but they are expressed through estimates of a nuisance parameter *ν* instead of *P*. For the sake of completeness we here give the exact construction of all estimators appearing in the main text. The estimators related to the ATE are given by

10 $$\begin{aligned} {\hat{\psi }}_j^{CF} = \sum _{k=1}^K \frac{n_k}{n} {\mathbb {P}}_n^k \varphi _j(\cdot ; {\hat{\nu }}_{-k}), \quad {\hat{\sigma }}_{\psi _j}^{2,CF} = \sum _{k=1}^K \frac{n_k}{n} {\mathbb {P}}_n^k\left( \varphi _j(\cdot ;{\hat{\nu }}_{-k}) - {\hat{\psi }}^{CF}\right) ^2. \end{aligned}$$

The Estimators of the target parameters related to the partially linear projection are given by

$$\begin{aligned} {\hat{\varGamma }}_j^{l, CF} = \frac{1}{n} \sum _{k=1}^K \sum _{i \in {\mathcal {T}}_k} \phi _{\varGamma _j^l}(O_i; {\hat{\nu }}_{l,-k}^2), \quad {\hat{\chi }}^{l, CF} = \frac{1}{n} \sum _{k=1}^K \sum _{i \in {\mathcal {T}}_k} \phi _{\chi ^l}(O_i; {\hat{\nu }}_{l,-k}^1), \quad {\hat{\varOmega }}_j^{l, CF} = \frac{{\hat{\varGamma }}_j^{l, CF}}{{\hat{\chi }}^{l, CF}}. \end{aligned}$$

As in the ATE setting, the variance, $P{\tilde{\psi }}^2_{\varOmega _j^l}$, is estimated by the cross-fitted plugin estimator:

$${\hat{\sigma }}_{\varOmega _j^l}^{2,CF} = \sum _{k=1}^K \frac{n_k}{n} {\mathbb {P}}_n^k{\tilde{\psi }}_{\varOmega _j^l}(\cdot ;{\hat{\nu }}^2_{l,-k})^2,$$

where (with some abuse of notation) we define

$${\tilde{\psi }}_{\varOmega _j^l}(O;{\hat{\nu }}^2_{l,-k}) = \frac{1}{{\hat{\chi }}^{l, CF}}\left( \phi _{\varGamma _j^l}(O; {\hat{\nu }}_{l,-k}^2) - {\hat{\varGamma }}_j^{l,CF} - {\hat{\varOmega }}_j^{l,CF} \left( \phi _{\chi ^l}(O; {\hat{\nu }}_{l,-k}^1) - {\hat{\chi }}^{l,CF} \right) \right) .$$

## Appendix B Identification of causal estimands

We present an argument for the identification results in the main text. The argument follows usual steps in the causal inference literature on censored data, and it is based of a combination of the G-formula (Robins 1986) and identification results from the literature on survival analysis (see e.g. Andersen et al. 1993; Martinussen 2006).

Since ${{\,\mathrm{\textrm{E}}\,}}Y_j(t^*) = \int _0^{t^*} F_j(s) \textrm{d}s$ we have that ${{\,\mathrm{\textrm{E}}\,}}(Y_j(t^*) \mid A=a, X=x) = \int _0^{t^*} F_j(s\mid a,x) \textrm{d}s$ which is identified in the observed data under assumption A4 and A3 (Andersen et al. 1993). For the ATE, this allows us to write

$$\begin{aligned}&E_0\{Y_j^1(t^*) - Y_j^0(t^*)\} \\&= E_0\{{{\,\mathrm{\textrm{E}}\,}}(Y_j^1(t^*) - Y_j^0(t^*)\mid X)\} \\&\overset{ass. A2}{=}\ E_0\{{{\,\mathrm{\textrm{E}}\,}}(Y_j^1(t^*)\mid A = 1, X) - {{\,\mathrm{\textrm{E}}\,}}(Y_j^0(t^*)\mid A = 0, X)\} \\&\overset{ass. A1}{=}\ E_0\{{{\,\mathrm{\textrm{E}}\,}}(Y_j(t^*)\mid A = 1, X) - {{\,\mathrm{\textrm{E}}\,}}(Y_j(t^*)\mid A = 0, X)\} \\&\overset{ass. A4}{=}\ E_0\{ L_j(0,t^*\mid A=1, X) - L_j(0,t^*\mid A=0, X)\} \end{aligned}$$

where assumption A3 ensures that all conditional distributions are well defined. We note that CATE is identified by the same arguments.

## Appendix C Derivation of influence functions

In the following we consider the parametric submodel $P_\epsilon = \epsilon Q + (1-\epsilon )P$, where *Q* is the Dirac measure with pointmass in the observation $O = ({\tilde{T}}, {\tilde{\varDelta }}, A, X)$, and we define the operator $\partial _\epsilon$ with $\partial _\epsilon f_\epsilon = \frac{d}{d \epsilon }|_{\epsilon = 0} f_\epsilon$.

### Lemma C1

The Gateaux derivative of $\varLambda _j(ds\mid a, x)$ is given by

11 $$\begin{aligned} \partial _\epsilon \varLambda _{j,\epsilon }(ds\mid a, x) = \frac{1}{P({\tilde{T}} \ge s, a, x)}\left( Q(ds, \varDelta = j, a, x) - \mathbb {1}({\tilde{T}}\ge s, a, x)\varLambda _j(ds\mid a, x)\right) \end{aligned}$$

where

$$P({\tilde{T}} \ge s, a, x) = \sum _{\delta = 0}^2\int _s^\infty P(ds, \delta , a, x).$$

### Proof

Observe

$$\begin{aligned}\partial _\epsilon \varLambda _{j,\epsilon }&(ds\mid a, x) \\ =&\partial _\epsilon \frac{P_\epsilon (ds, \varDelta = j, a, x)}{P_\epsilon ({\tilde{T}}\ge s, a, x)} \\ =&\frac{Q(ds, \varDelta = j, a, x) - P(ds, \varDelta = j, a, x)}{P({\tilde{T}}\ge s, a, x)} - \partial _\epsilon P_\epsilon ({\tilde{T}}\ge s, a, x) \frac{P(ds, \varDelta = j, a, x)}{P({\tilde{T}}\ge s, a, x)^2} \\ =&\frac{Q(ds, \varDelta = j, a, x) - P(ds, \varDelta = j, a, x)}{P({\tilde{T}}\ge s, a, x)} \\&- \sum _{\delta = 0}^2\left( \mathbb {1}({\tilde{T}}\ge s, \delta , a, x) - P({\tilde{T}}\ge s, \delta , a, x) \right) \frac{P(ds, \varDelta = j, a, x)}{P({\tilde{T}}\ge s, a, x)^2} \\ =&\frac{1}{P({\tilde{T}}\ge s, a, x)}\left( Q(ds, \varDelta = j, a, x) - \mathbb {1}({\tilde{T}}\ge s, a, x)\varLambda _j(ds\mid a, x) \right) \end{aligned}$$

*□*

### Lemma C2

The Gateaux derivative of *S(s∣ a,x)* is given by

12 $$\begin{aligned} \partial _\epsilon S_\epsilon (s\mid a, x) = -S(s\mid a, x)\frac{\mathbb {1}(A=a, X=x)}{\pi (a\mid x), f(x)} \int _0^s \frac{\textrm{d}M_1(u\mid a, x) + \textrm{d}M_2(u\mid a,x)}{P({\tilde{T}}\ge u, a, x)} \end{aligned}$$

### Proof

First we note that

13 $$\begin{aligned} \partial _\epsilon \varLambda _{j, \epsilon }(s\mid a, x) =&\int _0^s \partial _\epsilon \varLambda _{j,\epsilon }(ds\mid a, x) \nonumber \\ =&\frac{\mathbb {1}(A=a, X=x)}{\pi (a\mid x)f(x)} \int _0^s \frac{\textrm{d}M_j(u\mid a, x)}{P({\tilde{T}}\ge s\mid a, x)} \end{aligned}$$

by lemma C1. Then

$$\begin{aligned} \partial _\epsilon S(s\mid a, x)&= \partial _\epsilon \exp (-\left[ \varLambda _{1, \epsilon }(s\mid a,x) + \varLambda _{2, \epsilon }(s\mid a,x) \right] ) \\&= -S(s\mid a, x) \partial _\epsilon \left[ \varLambda _{1, \epsilon }(s\mid a,x) + \varLambda _{2, \epsilon }(s\mid a,x) \right] . \end{aligned}$$

Applying (13) gives the result. *□*

### Lemma C3

The Gateaux derivative of $L_j(0,t^*\mid a, x)$ is given by

14 $$\begin{aligned} \partial _\epsilon L_{j, \epsilon }(0,t^*\mid a, x) = \frac{\mathbb {1}(A=a, X=x)}{\pi (a\mid x)f(x)} \sum _{i=1,2} \int _0^{t^*} \frac{H_{ij}(s,t^*\mid a, x)}{S_C(s\mid a, x)} \textrm{d}M_i(s\mid a, x) \end{aligned}$$

where

$$H_{ij}(s,t\mid a,x) = \int _s^t \mathbb {1}(i=j) + \frac{F_j(s\mid a, x) - F_j(u\mid a, x)}{S(s\mid a, x)} \textrm{d}u.$$

### Proof

First we note that

15 $$\begin{aligned} \partial _\epsilon F_{j, \epsilon }(t\mid a, x) = \int _0^t \partial _\epsilon S_\epsilon (s\mid a, x)\varLambda _j(ds\mid a, x) + \int _0^t \partial _\epsilon S(s\mid a, x)\varLambda _{j, \epsilon }(ds\mid a, x). \end{aligned}$$

For the first term in (15), observe

16 $$\begin{aligned}\int _0^t &\partial _\epsilon S_\epsilon (s\mid a, x)\varLambda _j(ds\mid a, x) \nonumber \\ =&\frac{ \mathbb {1}(A=a, X=x)}{\pi (a\mid x)f(x)}\left[ \int _0^t \int _0^s \frac{-S(s\mid a, x)}{P({\tilde{T}}\ge u \mid a, x)} \textrm{d}M_1(u\mid a, x)\varLambda _j(s\mid a, x) \right. \nonumber \\&\left. \phantom {\frac{ \mathbb {1}(A=a, X=x)}{\pi (a\mid x)f(x)}} + \int _0^t \int _0^s \frac{-S(s\mid a, x)}{P({\tilde{T}}\ge u \mid a, x)}\textrm{d}M_2(u\mid a, x)\varLambda _j(s\mid a, x) \right] \end{aligned}$$

by lemma C2, and for the second term

17 $$\begin{aligned}\int _0^t &\partial _\epsilon S(s\mid a, x)\varLambda _{j, \epsilon }(ds\mid a, x) \nonumber \\ =&\int _0^t \frac{S(s\mid a,x)}{P({\tilde{T}} \ge s, a, x)}\left( Q(ds, \varDelta = j, a, x) - \mathbb {1}({\tilde{T}}\ge s, a, x)\varLambda _j(ds\mid a, x)\right) \nonumber \\ =&\frac{\mathbb {1}(A=a, X=x)}{\pi (a\mid x)f(x)}\int _0^t \frac{\textrm{d}N_j(s)}{S_C(s\mid a,x)} - \frac{\mathbb {1}({\tilde{T}}\ge s) \varLambda _j(ds\mid a, x)}{S_C(s\mid a,x)} \nonumber \\ =&\frac{\mathbb {1}(A=a, X=x)}{\pi (a\mid x)f(x)} \int _0^t \frac{\textrm{d}M_j(s\mid a, x)}{S_C(s\mid a,x)} \end{aligned}$$

by lemma C1. Plugging into (15) gives

18 $$\begin{aligned}\partial _\epsilon F_{j, \epsilon }&(t\mid a, x) \nonumber \\ =&\frac{\mathbb {1}(A=a, X=x)}{\pi (a\mid x)f(x)} \left[ \int _0^t \frac{\int _u^t-S(s\mid a, x) \varLambda _j(ds\mid a, x)}{P({\tilde{T}}\ge u\mid a, x)} \textrm{d}M_1(u\mid a, x) \right. \nonumber \\&\phantom {\frac{\mathbb {1}(A=a, X=x)}{\pi (a\mid x)f(x)} } + \int _0^t \frac{\int _u^t-S(s\mid a, x) \varLambda _j(ds\mid a, x)}{P({\tilde{T}}\ge u\mid a, x)} \textrm{d}M_2(u\mid a, x) + \left. \int _0^t \frac{\textrm{d}M_j(s\mid a, x)}{S_C(s\mid a,x)} \right] \nonumber \\ =&\frac{\mathbb {1}(A=a, X=x)}{\pi (a\mid x)f(x)} \left[ \int _0^t \frac{F_j(u\mid a,x) - F_j(t\mid a, x)}{S(u\mid a,x)S_C(u\mid a,x)} \textrm{d}M_1(u\mid a, x) \right. \nonumber \\&\phantom {\frac{\mathbb {1}(A=a, X=x)}{\pi (a\mid x)f(x)} } + \int _0^t \frac{F_j(u\mid a,x) - F_j(t\mid a, x)}{S(u\mid a,x)S_C(u\mid a,x)} \textrm{d}M_2(u\mid a, x) + \left. \int _0^t \frac{\textrm{d}M_j(u\mid a, x)}{S_C(u\mid a,x)} \right] \nonumber \\ =&\frac{\mathbb {1}(A=a, X=x)}{\pi (a\mid x)f(x)} \sum _{i=1,2} \int _0^t \frac{1}{S_C(u\mid a,x)} \left( \frac{F_j(u\mid a,x) - F_j(t\mid a, x)}{S(u\mid a,x)} + \mathbb {1}(i=j) \right) \textrm{d}M_i(u\mid a, x). \end{aligned}$$

Finally, by (18), we have

19 $$\begin{aligned}\partial _\epsilon L_{j, \epsilon }&(0, t^*\mid a, x) \nonumber \\ =&\int _0^{t^*} \partial _\epsilon F_{j, \epsilon }(t\mid a, x) \textrm{d}t \nonumber \\ =&\int _0^{t^*} \sum _{i=1,2} \int _0^t \frac{1}{S_C(u\mid a,x)} \left( \frac{F_j(u\mid a,x) - F_j(t\mid a, x)}{S(u\mid a,x)} + \mathbb {1}(i=j) \right) \textrm{d}M_i(u\mid a, x) \textrm{d}t \nonumber \\&\quad \times \frac{\mathbb {1}(A=a, X=x)}{\pi (a\mid x)f(x)} \nonumber \\ =&\int _0^{t^*} \sum _{i=1,2} \int _u^{t^*} \left( \frac{F_j(u\mid a,x) - F_j(t\mid a, x)}{S(u\mid a,x)} + \mathbb {1}(i=j) \right) \textrm{d}t\frac{1}{S_C(u\mid a,x)} \textrm{d}M_i(u\mid a, x) \nonumber \\&\times \frac{\mathbb {1}(A=a, X=x)}{\pi (a\mid x)f(x)} \nonumber \\ =&\frac{\mathbb {1}(A=a, X=x)}{\pi (a\mid x)f(x)} \sum _{i=1,2} \int _0^{t^*} \frac{H_{ij}(s,t^*\mid a, x)}{S_C(s\mid a, x)} \textrm{d}M_i(s\mid a, x). \end{aligned}$$

*□*

### Proof

(Proof of lemma 1) We have

$$\partial _\epsilon \psi _j(P_\epsilon ) = \partial _\epsilon {{\,\mathrm{\textrm{E}}\,}}_{P_\epsilon }\{ \tau _{j,P_\epsilon }(X)\} = \tau _j(X) + {{\,\mathrm{\textrm{E}}\,}}\{\partial _\epsilon \tau _{j,P_\epsilon }(X) \} - \psi _j(P).$$

Applying lemma C3 gives the result. *□*

### Proof

(Proof of lemma 2) The EIF of $\chi ^l(P)$ is given by theorem 3 in Ziersen and Martinussen (2025). By remark 2 in Ziersen and Martinussen (2025), the EIF of $\varGamma _j^l(P)$ is given by ${\tilde{\psi }}_{\varGamma _j^l}$ provided that the CATE function $\tau _j(x)$ has Gateaux derivative given by $\frac{\mathbb {1}(X=x)}{f(x)}\left[ \varphi _j(O;\nu ) - \tau _j(X) \right]$, which is seen to hold by lemma C3. The EIF of $\varOmega _j^l$ then follows from the chain rule. *□*

## Appendix D Proof of asymptotic results

In the following, we let $\tau ^a_j(X) = L_j(0,t^* \mid a, X)$, *a=0,1*, such that $\tau _j = \tau _j^1 - \tau _j^0$. Similarly, we define

$$\varphi ^a_j(O;\nu ) = \tau _j^a(X) + \frac{\mathbb {1}(A=a)}{\pi (a\mid X)}\biggl \{\sum _{i=1,2} \int _0^{t^*} \frac{H_{ij}(s,t^*\mid a, X)}{S_C(s\mid a, X)} \textrm{d}M_i(s\mid a, X)\biggr \},$$

*a=0,1*, such that $$\varphi _j = \varphi ^1_j - \varphi ^0_j$$.

### Lemma D1

The remainder term $P\{\varphi _j^a(\cdot ;{\hat{\nu }}) - \tau _j^a\}$, *a=0,1*, can be represented as

$$\begin{aligned}&{{\,\mathrm{\textrm{E}}\,}}\left\{ \sum _{i=1,2} \int _0^{t^*} S(s\mid a, X) {\hat{H}}_{ij}(s, t^*\mid a, X) \right. \\&\left. \quad \quad \times \left( 1 - \frac{\pi (a\mid X)S_C(s\mid a, X)}{{\hat{\pi }}(a\mid X){\hat{S}}_C(s\mid a, X)} \right) \textrm{d}\left[ {\hat{\varLambda }}_i(s\mid a, X) - \varLambda _i(s\mid a, X) \right] \right\} \end{aligned}$$

where the expectation is taken with respect to an observation *O*, considering the nuisance estimators, ${\hat{\nu }}$, fixed. Under assumption B2 it holds that $P\{\varphi _j(\cdot ;{\hat{\nu }}) - \tau _j\} = o_p(n^{-1/2})$.

### Proof

Throughout the proof, expectations and conditional expectations will be taken with respect to an observation *O*, considering estimated nuisance parameters fixed. For the first statement we have

20 $$\begin{aligned} P(\varphi _j^a(\cdot ;{\hat{\nu }}) - \tau _j^a) =&{{\,\mathrm{\textrm{E}}\,}}\left\{ {\hat{L}}_j(0, t^*\mid a, X) - L_j(0, t^*\mid a, X)\right\} \nonumber \\&+ {{\,\mathrm{\textrm{E}}\,}}\left\{ \frac{\mathbb {1}(A=a)}{{\hat{\pi }}(a\mid X)} \sum _{i = 1,2} \int _0^{t^*} \frac{{\hat{H}}_{ij}(s, t^*\mid a, X)}{{\hat{S}}_C(s\mid a, X)} \textrm{d}{\hat{M}}_i(s\mid a, X) \right\} \end{aligned}$$

and we will expand the two terms separately. For the first term note that

21 $$\begin{aligned} {\hat{F}}_j(t \mid a, x) - F_j(t\mid a, x) =&\int _0^t {\hat{S}}(s\mid a, x) - S(s\mid a, x) \textrm{d}{\hat{\varLambda }}_j(s\mid a,x) \nonumber \\&+ \int _0^t S(s\mid a,x) \left[ {\hat{\varLambda }}_j(s\mid a,x) - \varLambda _j(s\mid a,x) \right] . \end{aligned}$$

Define

$$\varLambda (s\mid a,x) = \varLambda _1(s\mid a, x) + \varLambda _1(s\mid a, x),$$

then Duhamel’s equations (Gill and Johansen 1990) gives

$${\hat{S}}(s\mid a, x) - S(s\mid a, x) = \int _0^s \frac{S(u\mid a, x)}{{\hat{S}}(u\mid a,x)} \textrm{d}\left[ \varLambda (u\mid a,x ) - {\hat{\varLambda }}(u\mid a,x ) \right] {\hat{S}}(s\mid a, x).$$

Plugging this into the first term in (21) gives

$$\begin{aligned}\int _0^t &{\hat{S}}(s\mid a, x) - S(s\mid a, x) \textrm{d}{\hat{\varLambda }}_j(s\mid a,x) \\ =&\int _0^t \int _0^s \frac{S(u\mid a, x)}{{\hat{S}}(u\mid a,x)} \textrm{d}\left[ \varLambda (u\mid a,x ) - {\hat{\varLambda }}(u\mid a,x ) \right] {\hat{S}}(s\mid a, x) \textrm{d}{\hat{\varLambda }}_j(s\mid a,x) \\ =&\int _0^t \int _u^t {\hat{S}}(s\mid a, x) \frac{S(u\mid a, x)}{{\hat{S}}(u\mid a,x)} \textrm{d}{\hat{\varLambda }}_j(s\mid a,x) \textrm{d}\left[ \varLambda (u\mid a,x ) - {\hat{\varLambda }}(u\mid a,x ) \right] \\ =&\int _0^t \frac{S(u\mid a, x)}{{\hat{S}}(u\mid a,x)} \left( {\hat{F}}_j(t\mid a,x) - {\hat{F}}_j(u\mid a,x) \right) \textrm{d}\left[ \varLambda (u\mid a,x ) - {\hat{\varLambda }}(u\mid a,x ) \right] \\ =&\int _0^t \frac{S(u\mid a, x)}{{\hat{S}}(u\mid a,x)} \left( {\hat{F}}_j(t\mid a,x) - {\hat{F}}_j(u\mid a,x) \right) \textrm{d}\left[ \varLambda _1(u\mid a,x ) - {\hat{\varLambda }}_1(u\mid a,x ) \right] \\&+ \int _0^t \frac{S(u\mid a, x)}{{\hat{S}}(u\mid a,x)} \left( {\hat{F}}_j(t\mid a,x) - {\hat{F}}_j(u\mid a,x) \right) \textrm{d}\left[ \varLambda _2(u\mid a,x ) - {\hat{\varLambda }}_2(u\mid a,x ) \right] \end{aligned}$$

Using this expansion, we can write (21) as

22 $$\begin{aligned}{\hat{F}}_j&(t \mid a, x) - F_j(t\mid a, x) \nonumber \\ =&\int _0^t \frac{S(u\mid a, x)}{{\hat{S}}(u\mid a,x)} \left( {\hat{F}}_j(u\mid a,x) - {\hat{F}}_j(t\mid a,x) \right) \textrm{d}\left[ {\hat{\varLambda }}_1(u\mid a,x ) - \varLambda _1(u\mid a,x) \right] \nonumber \\&+ \int _0^t \frac{S(u\mid a, x)}{{\hat{S}}(u\mid a,x)} \left( {\hat{F}}_j(u\mid a,x) - {\hat{F}}_j(t\mid a,x) \right) \textrm{d}\left[ {\hat{\varLambda }}_2(u\mid a,x ) - \varLambda _2(u\mid a,x) \right] \nonumber \\&+ \int _0^t S(s\mid a,x) \textrm{d}\left[ {\hat{\varLambda }}_j(s\mid a,x) - \varLambda _j(s\mid a,x) \right] \nonumber \\ =&\sum _{i=1,2}\int _0^t S(s\mid a,x) \left( \mathbb {1}(i = j) + \frac{{\hat{F}}_j(s\mid a,x) - {\hat{F}}_j(t\mid a,x)}{{\hat{S}}(s\mid a, x)} \right) \textrm{d}\left[ {\hat{\varLambda }}_i(s\mid a,x ) - \varLambda _i(s\mid a,x) \right] \end{aligned}$$

For the second term in (20) note that

23 $$\begin{aligned}{{\,\mathrm{\textrm{E}}\,}}&\left( \int _0^{t^*} \frac{{\hat{H}}_{ij}(s, t^*\mid A, X)}{{\hat{S}}_C(s\mid A, X)} \textrm{d}{\hat{M}}_i(s\mid A, X) \ \biggl | \ A, X \right) \nonumber \\ =&\int _0^{t^*} \frac{{\hat{H}}_{ij}(s, t^*\mid A, X)}{{\hat{S}}_C(s\mid A, X)} {{\,\mathrm{\textrm{E}}\,}}(\textrm{d}N_i(s) \mid A, X) \nonumber \\&- \int _0^{t^*} \frac{{\hat{H}}_{ij}(s, t^*\mid A, X)}{{\hat{S}}_C(s\mid A, X)} {{\,\mathrm{\textrm{E}}\,}}(\mathbb {1}({\tilde{T}}\ge s) \mid A, X) \textrm{d}{\hat{\varLambda }}_i(s\mid A, X) \nonumber \\ =&\int _0^{t^*} \frac{{\hat{H}}_{ij}(s, t^*\mid A, X)}{{\hat{S}}_C(s\mid A, X)} S(s\mid A, X) S_C(s\mid A, X) \textrm{d}\left[ \varLambda _i(s\mid A, X) - {\hat{\varLambda }}_i(s\mid A, X) \right] . \end{aligned}$$

Hence, by collecting (22) and (23), and using iterated expectation, we can write (20) as

24 $$\begin{aligned}{{\,\mathrm{\textrm{E}}\,}}&\left\{ \sum _{i=1,2}\int _0^{t^*}\int _0^u S(s\mid a,X) \left( \mathbb {1}(i = j) + \frac{{\hat{F}}_j(s\mid a,X) - {\hat{F}}_j(u\mid a,X)}{{\hat{S}}(s\mid a, X)} \right) \right. \nonumber \\&\phantom {\sum _{i=1,2}\int _0^{t^*}\int _0^u S(s\mid a,X) \sum _{i=1,2}\int _0^{t^*}\int _0^u S(s\mid a,X)} \times \textrm{d}\left[ {\hat{\varLambda }}_i(s\mid a,X ) - \varLambda _i(s\mid a,X) \right] \textrm{d}u \nonumber \\&\left. -\frac{\pi (a\mid X)}{{\hat{\pi }}(a\mid X)} \sum _{i=1,2} \int _0^{t^*} \frac{{\hat{H}}_{ij}(s, t^*\mid a, X)}{{\hat{S}}_C(s\mid a, X)} S(s\mid a, X) S_C(s\mid a, X) \textrm{d}\left[ {\hat{\varLambda }}_i(s\mid a, X) - \varLambda _i(s\mid a, X) \right] \right\} \nonumber \\ =&{{\,\mathrm{\textrm{E}}\,}}\left\{ \sum _{i=1,2} \int _0^{t^*} S(s\mid a, X) {\hat{H}}_{ij}(s,t^*\mid a, X) \right. \nonumber \\&\left. \quad \quad \times \left( 1 - \frac{\pi (a\mid X) S_C(s\mid a, X)}{{\hat{\pi }}(a\mid X) {\hat{S}}_C(s\mid a, X)} \right) \textrm{d}\left[ {\hat{\varLambda }}_i(s\mid a, X) - \varLambda _i(s\mid a, X) \right] \right\} \end{aligned}$$

which gives the first statement. For the second statement note that

$$P\{\varphi _j(\cdot ;{\hat{\nu }}) - \tau _j\} = P\{\varphi ^1_j(\cdot ;{\hat{\nu }}) - \tau ^1_j\} - P\{\varphi ^0_j(\cdot ;{\hat{\nu }}) - \tau ^0_j\}.$$

Applying the representation above along with assumption B2 gives the result. *□*

### Lemma D2

Let $g(s\mid a,x) = \pi (a\mid x) S_C(s\mid a,x)$ and ${\hat{g}}(s\mid a,x) = {\hat{\pi }}(a\mid x) {\hat{S}}_C(s\mid a,x)$. Under assumption B1 and B3, the uncentered EIF, $\varphi _j$, is bounded in $L_2(P)$-norm as:

$$\begin{aligned} \left\Vert \varphi _j^a(\cdot ;{\hat{\nu }}) - \varphi _j^a(\cdot ;\nu )\right\Vert \le&C_1^*\left\Vert \sup _{s\le t^*}\left| {\hat{\varLambda }}_1(s\mid a, X) - \varLambda _1(s\mid a, X) \right| \right\Vert \\&+ C_2^*\left\Vert \sup _{s\le t^*}\left| {\hat{\varLambda }}_2(s\mid a, X) - \varLambda _2(s\mid a, X) \right| \right\Vert \\&+ C_c^*\left\Vert \sup _{s\le t^*}\left| {\hat{g}}(s\mid a, X) - g(s\mid a, X) \right| \right\Vert . \end{aligned}$$

Furthermorre, $$\left\Vert \varphi _j(\cdot ;{\hat{\nu }}) - \varphi _j(\cdot ;\nu )\right\Vert = o_p(1).$$

### Proof

Observe that

25 $$\begin{aligned}P&\{\varphi _j^a(\cdot ;{\hat{\nu }}) - \varphi _j^a(\cdot ;\nu )\}^2 \nonumber \\ \le&2{{\,\mathrm{\textrm{E}}\,}}\left\{ {\hat{L}}_j(0,t^*\mid a, X) - L_j(0,t^*\mid a, X) \right\} ^2 \end{aligned}$$

26 $$\begin{aligned}&+ 2{{\,\mathrm{\textrm{E}}\,}}\left\{ \sum _{i=1,2}\int _0^{t^*} \frac{{\hat{H}}_{ij}(s,t^*\mid a, X)}{{\hat{g}}(s\mid a, X)} - \frac{H_{ij}(s,t^*\mid a, X)}{g(s\mid a, X)} \textrm{d}N_i(s) \right\} ^2 \end{aligned}$$

27 $$\begin{aligned}&+ 2{{\,\mathrm{\textrm{E}}\,}}\left\{ \sum _{i=1,2}\left[ \int _0^{t^*\wedge {\tilde{T}}} \frac{{\hat{H}}_{ij}(s,t^*\mid a, X)}{{\hat{g}}(s\mid a, X)} \textrm{d}{\hat{\varLambda }}_i(s\mid a,X) - \int _0^{t^*\wedge {\tilde{T}}} \frac{H_{ij}(s,t^*\mid a, X)}{g(s\mid a, X)} \textrm{d}\varLambda _i(s\mid a, X) \right] \right\} ^2. \end{aligned}$$

We will deal with each of the terms separately, but first we will derive some results for the consistency of ${\hat{F}}_j$, ${\hat{L}}_j$ and ${\hat{H}}_{ij}$. For ${\hat{F}}_j$ we have that

28 $$\begin{aligned}{\hat{F}}_j&(t^*\mid a, x) - F_j(t^*\mid a, x) \nonumber \\ =&\int _0^{t^*} {\hat{S}}(s\mid a, x) \textrm{d}\left[ {\hat{\varLambda }}_j(s\mid a, x) - \varLambda _j(s\mid a, x) \right] + \int _0^{t^*} {\hat{S}}(s\mid a,x) - S(s\mid a,x) \textrm{d}\varLambda _j(s\mid a, x) \nonumber \\ =&{\hat{S}}(t^*\mid a,x)\left[ {\hat{\varLambda }}_j(t^* \mid a,x) - \varLambda _j(t^* \mid a,x) \right] - {\hat{S}}(0\mid a,x)\left[ {\hat{\varLambda }}_j(0 \mid a,x) - \varLambda _j(0 \mid a,x) \right] \nonumber \\&- \int _0^{t^*} {\hat{\varLambda }}_j(s\mid a, x) - \varLambda _j(s\mid a, x) \textrm{d}{\hat{S}}(s\mid a, x) + \int _0^{t^*} {\hat{S}}(s\mid a,x) - S(s\mid a,x) \textrm{d}\varLambda _j(s\mid a, x) \nonumber \\ \le&2\sup _{s\le t^*}\left| {\hat{\varLambda }}_j(s\mid a, x) - \varLambda _j(s\mid a, x) \right| + {\hat{\varLambda }}_j(t\mid a,x)\sup _{s\le t^*}\left| {\hat{S}}(s\mid a, x) - S(s\mid a, x) \right| \nonumber \\ \le&2\sup _{s\le t^*}\left| {\hat{\varLambda }}_j(s\mid a, x) - \varLambda _j(s\mid a, x) \right| \nonumber \\&+ \log (\eta ^{-1}) e^C \sup _{s\le t^*} \left[ \left| {\hat{\varLambda }}_1(s\mid a, x) - \varLambda _1(s\mid a, x) \right| + \left| {\hat{\varLambda }}_2(s\mid a, x) - \varLambda _2(s\mid a, x) \right| \right] \nonumber \\ =&C_1 \sup _{s\le t^*}\left| {\hat{\varLambda }}_1(s\mid a, x) - \varLambda _1(s\mid a, x) \right| + C_2 \sup _{s\le t^*}\left| {\hat{\varLambda }}_2(s\mid a, x) - \varLambda _2(s\mid a, x) \right| \end{aligned}$$

for constants $C_1>0$ and $C_2> 0$. The second equality follows from partial integration and the second inequality follows from the mean value theorem with *C>0* is some value between ${\hat{\varLambda }}(s\mid a,x)$ and $\varLambda (s\mid a,x)$ together with assumption B1.

From (28) it follows immediately that

29 $$\begin{aligned}{\hat{L}}_j&(0,t^*\mid a, x) - L_j(0,t^*\mid a, x) \nonumber \\ \le&t^*C_1 \sup _{s\le t^*}\left| {\hat{\varLambda }}_1(s\mid a, x) - \varLambda _1(s\mid a, x) \right| + t^*C_2 \sup _{s\le t^*}\left| {\hat{\varLambda }}_2(s\mid a, x) - \varLambda _2(s\mid a, x) \right| . \end{aligned}$$

For $${\hat{H}}_{ij}$$, observe

30 $$\begin{aligned}{\hat{H}}_{ij}&(s,t^*\mid a,x) - H_{ij}(s,t^*\mid a,x) \nonumber \\ =&\int _s^{t^*} \frac{{\hat{F}}_j(s\mid a,x) - F_j(s\mid , a, x) - ({\hat{F}}_j(u\mid a,x) - F_j(u\mid , a, x))}{{\hat{S}}(s\mid a,x)} \textrm{d}u \nonumber \\&+ \int _s^{t^*}\left( \frac{1}{{\hat{S}}(s\mid a,x)} - \frac{1}{S(s\mid a,x)} \right) \left( F_j(s\mid a,x) - F_j(u\mid a, x) \right) \textrm{d}u \nonumber \\ \le&t^* \left| \frac{{\hat{F}}_j(s\mid a,x) - F_j(s\mid a,x)}{{\hat{S}}(s\mid a,x)} \right| + \eta ^{-1}\int _s^{t^*}\left| {\hat{F}}_j(u\mid a,x) - F_j(u\mid a,x) \right| \textrm{d}u \nonumber \\&+ \eta ^{-2} \left| {\hat{S}}(s\mid a,x) - S(s\mid a,x) \right| \left| F_j(s\mid a,x)(t^* - s) - \int _s^{t^*} F_j(u\mid a,x) \textrm{d}u \right| \nonumber \\ \le&C_3 \sup _{s\le t^*}\left| {\hat{\varLambda }}_1(s\mid a, x) - \varLambda _1(s\mid a, x) \right| + C_4 \sup _{s\le t^*}\left| {\hat{\varLambda }}_2(s\mid a, x) - \varLambda _2(s\mid a, x) \right| \end{aligned}$$

for some constants $C_3>0$ and $C_4> 0$, which follows from (28). Now we proceed to bound each of the three terms initially stated. The bound of (25) follows directly from (29):

31 $$\begin{aligned}&2{{\,\mathrm{\textrm{E}}\,}}\left\{ {\hat{L}}_j(0,t^*\mid a, X) - L_j(0,t^*\mid a, X) \right\} ^2 \nonumber \\ \le&2{{\,\mathrm{\textrm{E}}\,}}\left\{ C_5 \sup _{s\le t^*}\left| {\hat{\varLambda }}_1(s\mid a, x) - \varLambda _1(s\mid a, x) \right| + C_6 \sup _{s\le t^*}\left| {\hat{\varLambda }}_2(s\mid a, x) - \varLambda _2(s\mid a, x) \right| \right\} ^2. \end{aligned}$$

For (26) we have

32 $$\begin{aligned}{{\,\mathrm{\textrm{E}}\,}}&\left\{ \sum _{i=1,2}\int _0^{t^*} \frac{{\hat{H}}_{ij}(s,t^*\mid a, X)}{{\hat{g}}(s\mid a, X)} - \frac{H_{ij}(s,t^*\mid a, X)}{g(s\mid a, X)} \textrm{d}N_i(s) \right\} ^2 \nonumber \\ =&{{\,\mathrm{\textrm{E}}\,}}\left\{ \sum _{i=1,2} \int _0^{t^*} \frac{{\hat{H}}_{ij}(s,t^*\mid a, X) - H_{ij}(s,t^*\mid a, X)}{{\hat{g}}(s\mid a, X)} \textrm{d}N_i(s) \right. \nonumber \\&+ \left. \int _0^{t^*} H_{ij}(s,t^*\mid a,x) \left( \frac{1}{{\hat{g}}(s\mid a,X)} - \frac{1}{g(s\mid a,X)} \right) \textrm{d}N_i(s) \right\} ^2 \nonumber \\ \le&{{\,\mathrm{\textrm{E}}\,}}\left\{ \sum _{i=1,2} \eta ^{-2} \sup _{s\le t^*}\left| {\hat{H}}_{ij}(s\mid a,X) - H_{ij}(s\mid a,X) \right| \right. \end{aligned}$$

33 $$\begin{aligned}&\left. + \eta ^{-4}\sup _{s\le t^*} \left| {\hat{g}}(s\mid a,X) - g(s\mid a,X) \right| \left| H_{ij}(s,t^*\mid a,X) \right| \right\} ^2 \nonumber \\ \le&{{\,\mathrm{\textrm{E}}\,}}\left\{ C_7 \sup _{s\le t^*}\left| {\hat{\varLambda }}_1(s\mid a, X) - \varLambda _1(s\mid a, X) \right| + C_8 \sup _{s\le t^*}\left| {\hat{\varLambda }}_2(s\mid a, X) - \varLambda _2(s\mid a, X) \right| \right. \nonumber \\&\left. \phantom {{{\,\mathrm{\textrm{E}}\,}}\{ \left| {\hat{\varLambda }} \right| } + C_9 \sup _{s\le t^*} \left| {\hat{g}}(s\mid a,X) - g(s\mid a,X) \right| \right\} ^2. \end{aligned}$$

The first inequality follows from assumption B1 and the second inequality follows from (30) together with

34 $$\begin{aligned} H_{ij}(s,t^*\mid a,x) \le&\eta ^{-1}\int _s^{t^*}\left| F_j(s) - F_j(u) \right| \textrm{d}u + t^* - s \nonumber \\ \le&\eta ^{-1} 2 t^* \end{aligned}$$

by assumption B1.

Lastly, for (27) we have

35 $$\begin{aligned}{{\,\mathrm{\textrm{E}}\,}}&\left\{ \sum _{i=1,2}\left[ \int _0^{t^*\wedge {\tilde{T}}} \frac{{\hat{H}}_{ij}(s,t^*\mid a, X)}{{\hat{g}}(s\mid a, X)} \textrm{d}{\hat{\varLambda }}_i(s\mid a,X) - \int _0^{t^*\wedge {\tilde{T}}} \frac{H_{ij}(s,t^*\mid a, X)}{g(s\mid a, X)} \textrm{d}\varLambda _i(s\mid a, X) \right] \right\} ^2 \nonumber \\ =&E\left\{ \underbrace{\sum _{i=1,2} \left[ \int _0^{t^*\wedge {\tilde{T}}} {\hat{H}}_{ij}(s,t^*\mid a, X) \left( \frac{1}{{\hat{g}}(s\mid a,x)} - \frac{1}{g(s\mid a,x)} \right) \textrm{d}{\hat{\varLambda }}_i(s\mid a,x) \right] }_{(a)} \right. \nonumber \\&\left. + \underbrace{ \int _0^{t^*\wedge {\tilde{T}}} \sum _{i=1,2} \frac{{\hat{H}}_{ij}(s,t^*\mid a, X)}{g(s\mid a,X)} \textrm{d}{\hat{\varLambda }}_i(s\mid a,X) - \int _0^{t^*\wedge {\tilde{T}}} \sum _{i=1,2} \frac{H_{ij}(s\mid a, X)}{g(s\mid a,X)} \textrm{d}\varLambda _i(s\mid a,X)}_{(b)} \right\} ^2, \end{aligned}$$

and we will bound (*a*) and (*b*) separately. For (*a*) we have that for almost all *x*

36 $$\begin{aligned}\int _0^{t^*\wedge {\tilde{T}}}& {\hat{H}}_{ij}(s, t^*\mid a, x) \left( \frac{1}{{\hat{g}}(s\mid a,x)} - \frac{1}{g(s\mid a,x)} \right) \textrm{d}{\hat{\varLambda }}_i(s\mid a,x) \nonumber \\ \le&\sum _{i=1,2} \sup _{s\le t^*}\left| {\hat{H}}_{ij}(s, t^* \mid a, x)\right| \sup _{s\le t^*}\left| \frac{1}{{\hat{g}}(s\mid a,x)} - \frac{1}{g(s\mid a,x)} \right| {\hat{\varLambda }}_i(t^*\mid a,x) \nonumber \\ \le&4\eta ^{-5}t^*\log (\eta ) \sup _{s\le t^*}\left| {\hat{g}}(s\mid a,x) - g(s\mid a,x) \right| . \end{aligned}$$

by (34) together with assumption B1. Next, to control (b), define

$$H_j(s, t^* \mid a,x) = \int _s^{t^*} \frac{F_j(s\mid a,x) - F_j(u\mid a,x)}{S(s\mid a, x)} \textrm{d}u$$

and let

$$\begin{aligned} \varLambda (s\mid a,x) = \varLambda _1(s\mid a,x) + \varLambda _2(s\mid a,x) \end{aligned}$$

such that $S(s\mid a,x) = e^{-\varLambda (s\mid a,x)}$. Observe that

$$\begin{aligned}H_j&(s,t^*\mid a,x) \\ =&- \int _s^{t^*} \frac{F_j(u\mid a,x) - F_j(s\mid a,x)}{S(s\mid a,x)} \textrm{d}u \\ =&-\int _s^{t^*} \int _s^u \frac{S(v\mid a,x)}{S(s\mid a,x)} \varLambda _j(dv\mid a,x) \textrm{d}u \\ \overset{(*)}{=}&\ - \int _s^{t^*} \int _s^u \left( 1 - \int _s^v \frac{S(v\mid a,x)}{S(w\mid a,x)} \varLambda (dw\mid a,x) \right) \varLambda _j(dv\mid a,x) \textrm{d}u \\ =&- \left[ \int _s^{t^*} \int _s^u \varLambda _j(\textrm{d}v\mid a,x) \textrm{d}u - \int _s^{t^*} \int _s^u \int _s^v \frac{S(v\mid a,x)}{S(w\mid a,x)} \varLambda (\textrm{d}w\mid a,x) \varLambda _j(\textrm{d}v\mid a,x) \textrm{d}u \right] \\ =&- \left[ \int _s^{t^*} \int _v^{t^*} \textrm{d}u \varLambda _j(\textrm{d}v\mid a,x) - \int _s^{t^*} \int _w^{t^*} \int _w^u \frac{S(v\mid a,x)}{S(w\mid a,x)} \varLambda _j(dv\mid a,x) \textrm{d}u \varLambda (dw\mid a,x) \right] \\ =&- \left[ \int _s^{t^*} \int _v^{t^*} \textrm{d}u \varLambda _j(\textrm{d}v\mid a,x) - \int _s^{t^*} \int _w^{t^*} \frac{F_j(u\mid a,x) - F_j(w\mid a,x)}{S(w\mid a,x)} \textrm{d}u \varLambda (dw\mid a,x) \right] \\ =&- \int _s^{t^*} \sum _{i=1,2} H_{ij}(w,t^*\mid a,x) \varLambda _i(\textrm{d}w\mid a,x) \end{aligned}$$

where *(*)* follows from the backward equation (theorem 5, Gill and Johansen 1990). Hence

$$\begin{aligned} H_j(\textrm{d}s,t^*\mid a,x) = \sum _{i=1,2} H_{ij}(s,t^*\mid a,x) \varLambda _i(\textrm{d}s\mid a,x). \end{aligned}$$

From this expression we can derive a bound for (*b*) using integration by parts:

37 $$\begin{aligned}\int _0^{t^*\wedge {\tilde{T}}}& \sum _{i=1,2} \frac{{\hat{H}}_{ij}(s,t^*\mid a, x)}{g(s\mid a,x)} \textrm{d}{\hat{\varLambda }}_i(s\mid a,x) - \int _0^{t^*\wedge {\tilde{T}}} \sum _{i=1,2} \frac{H_{ij}(s\mid a, x)}{g(s\mid a,x)} \textrm{d}\varLambda _i(s\mid a,x) \nonumber \\ =&\int _0^{t^*\wedge {\tilde{T}}} \frac{1}{g(s\mid a,x)} \textrm{d}\left[ {\hat{H}}_j(s, t^*\mid a,x) - H_j(s, t^*\mid a,x) \right] \nonumber \\ =&\frac{1}{g(t^*\mid a,x)} \left[ {\hat{H}}_j(t^*\wedge {\tilde{T}}, t^*\mid a,x) - H_j(t^*\wedge {\tilde{T}}, t^*\mid a,x) \right] \nonumber \\&- \frac{1}{g(0\mid a,x)}\left[ {\hat{H}}_j(0, t^*\mid a,x) - H_j(0, t^*\mid a,x) \right] \nonumber \\&- \int _0^{t^*\wedge {\tilde{T}}} \left[ {\hat{H}}_j(s, t^*\mid a,x) - H_j(s, t^*\mid a,x) \right] \left( \frac{1}{g} \right) (ds\mid a,x) \nonumber \\ \le&3\eta ^{-1}\sup _{s\le t^*} \left| {\hat{H}}_j(s, t^*\mid a,x) - H_j(s, t^*\mid a,x) \right| \nonumber \\ \le&C_{10} \sup _{s\le t^*}\left| {\hat{\varLambda }}_1(s\mid a, x) - \varLambda _1(s\mid a, x) \right| + C_{11} \sup _{s\le t^*}\left| {\hat{\varLambda }}_2(s\mid a, x) - \varLambda _2(s\mid a, x) \right| \end{aligned}$$

for some constants $C_{10}>0$ and $C_{11}>0$. Applying the bounds (36) and (37) to (35) gives that (27) is bounded by

38 $$\begin{aligned}&E\left\{ C_{10} \sup _{s\le t^*}\left| {\hat{\varLambda }}_1(s\mid a, X) - \varLambda _1(s\mid a, X) \right| + C_{11} \sup _{s\le t^*}\left| {\hat{\varLambda }}_2(s\mid a, X) - \varLambda _2(s\mid a, X) \right| \right. \nonumber \\&+ \left. C_{12} \sup _{s\le t^*}\left| {\hat{g}}_c(s\mid a, X) - g(s\mid a, X) \right| \right\} ^2. \end{aligned}$$

Thus, applying (31), (32) and (38) to (25), (26) and (27), respectively, gives the first result. For the second result, note that

$$\left\Vert \varphi _j(\cdot ;{\hat{\nu }}) - \varphi _j(\cdot ;\nu )\right\Vert \le \left\Vert \varphi _j^1(\cdot ;{\hat{\nu }}) - \varphi _j^1(\cdot ;\nu )\right\Vert + \left\Vert \varphi _j^0(\cdot ;{\hat{\nu }}) - \varphi _j^0(\cdot ;\nu )\right\Vert .$$

Hence, the first result combined with assumption B3 gives the second result. *□*

### Proof

(Proof of Theorem 1) Consider the decomposition

$$\begin{aligned} {\mathbb {P}}_n^k \varphi _j(\cdot ;{\hat{\nu }}_{-k}) = {\mathbb {P}}_n^k{\tilde{\psi }}_{\psi _j} + ({\mathbb {P}}_n^k - P)(\varphi _j(\cdot ;{\hat{\nu }}_{-k}) - \varphi _j(\cdot ;\nu )) + P\varphi _j(\cdot ;{\hat{\nu }}_{-k}) \end{aligned}$$

such that

$$\begin{aligned} {\hat{\psi }}_j^{CF} - \psi _j&= \sum _{k=1}^K \frac{n_k}{n}{\mathbb {P}}_n^k\varphi _j(\cdot ;{\hat{\nu }}_{-k}) - \psi _j \\&= {\mathbb {P}}_n{\tilde{\psi }}_{\psi _j} + \underbrace{\sum _{k=1}^K \frac{n_k}{n}({\mathbb {P}}_n^k - P)(\varphi _j(\cdot ;{\hat{\nu }}_{-k}) - \varphi _j(\cdot ;\nu ))}_{\text {empirical process term}} + \underbrace{\sum _{k=1}^K \frac{n_k}{n} P(\varphi _j(\cdot ;{\hat{\nu }}_{-k}) - \psi _j)}_{\text {remainder term}}. \end{aligned}$$

Now, if both the empirical process term and the remainder term in the above display are $o_p(n^{-1/2})$, it follows that ${\hat{\psi }}_j^{CF}$ is asymptotically linear with influence function given by ${\tilde{\psi }}_j$. By Proposition 2 in Kennedy (2022) this is achieved if $\left\Vert \varphi _j(\cdot ;{\hat{\nu }}_{-k}) - \varphi _j(\cdot ;\nu )\right\Vert =o_p(1)$ for each *k* and if the remainder is $o_p(n^{-1/2})$. Under assumption B for each *k*, the former is achieved by lemma D2 and the latter is achieved by lemma D1. An application of the central limit theorem together with Slutsky’s lemma gives the convergence in distribution. *□*

### Proof

(Proof of Theorem 2) We will show that ${\hat{\varGamma }}_j^{l,CF}$ and ${\hat{\chi }}_j^{l,CF}$ are asymptotically linear with influence function given by ${\tilde{\psi }}_{\varGamma _j^l}$ and ${\tilde{\psi }}_{\chi _j^l}$, respectively. Since ${\hat{\varOmega }}_j^{l,CF}$ is a ratio of the two, it follows from the functional delta method that it is asymptotically linear with influence function given by ${\tilde{\psi }}_{\varOmega _j}$ (Van Der Vaart 2000, Ch. 25.7).

That ${\hat{\chi }}^{l,CF}$ is asymptotically linear follows from Theorem 5 in Ziersen and Martinussen (2025). To show that ${\hat{\varGamma }}_j^{l,CF}$ is asymptotically linear, we consider the decomposition

$$\begin{aligned} {\mathbb {P}}_n^k \phi _{\varGamma _j^l}(\cdot ;{\hat{\nu }}^2_{-k}) = {\mathbb {P}}_n^k{\tilde{\psi }}_{\varGamma _j^l} + ({\mathbb {P}}_n^k - P)(\phi _{\varGamma _j^l}(\cdot ;{\hat{\nu }}^2_{-k}) - \phi _{\varGamma _j^l}(\cdot ;\nu ^2)) + P\phi _{\varGamma _j^l}(\cdot ;{\hat{\nu }}^2_{-k}) \end{aligned}$$

such that

$$\begin{aligned} {\hat{\varGamma }}_j^{l,CF} - \Gamma_j^l &= \sum _{k=1}^K \frac{n_k}{n}{\mathbb {P}}_n^k\phi _{\varGamma _j^l}(\cdot ;{\hat{\nu }}^2_{-k}) - \varGamma _j^l \\&= {\mathbb {P}}_n{\tilde{\psi }}_{\varGamma _j^l} + \underbrace{\sum _{k=1}^K \frac{n_k}{n}({\mathbb {P}}_n^k - P)(\phi _{\varGamma _j^l}(\cdot ;{\hat{\nu }}^2_{-k}) - \phi _{\varGamma _j^l}(\cdot ;\nu ^2))}_{\text {empirical process term}} + \underbrace{\sum _{k=1}^K \frac{n_k}{n} P(\phi _{\varGamma _j^l}(\cdot ;{\hat{\nu }}^2_{-k}) - \varGamma _j^l)}_{\text {remainder term}}. \end{aligned}$$

Again, by Proposition 2 in Kennedy (2022), the desired asymptotic linearity follows if we can show that $\left\Vert \phi _{\varGamma _j^l}(\cdot ;{\hat{\nu }}^2_{-k}) - \phi _{\varGamma _j^l}(\cdot ;\nu ^2)\right\Vert = o_p(1)$ for each *k* and that the remainder term is $o_p(n^{-1/2})$. For both results, we will consider arguments that are similar to the ones given in the proof of Theorem 4 in Ziersen and Martinussen (2025).

*Empirical process term*

Consider the following expansion for a given *k*:

$$\begin{aligned} \phi _{\varGamma _j^l}(O;{\hat{\nu }}^2_{-k}) - \phi _{\varGamma _j^l}(O;\nu ^2) =&[\varphi _j(O;{\hat{\nu }}_{-k}) - \varphi _j(O;\nu )][X_l - {\hat{E}}^l_{-k}(X_{-l})] \\&- [{\hat{\tau }}_{j,-k}^l(X_{-l}) - \tau _j^l(X_{-l})][X_l - {\hat{E}}_{-k}^l(X_{-l})] \\&- [{\hat{E}}_{-k}^l(X_{-l}) - E(X_l\mid X_{-l})][\varphi _j(O;\nu ) - \tau _j^l(X_{-l})]. \end{aligned}$$

For the first term we have

$${{\,\mathrm{\textrm{E}}\,}}\left\{ [\varphi _j(O;{\hat{\nu }}_{-k}) - \varphi _j(O;\nu )][X_l - {\hat{E}}^l_{-k}(X_{-l})]\right\} ^2 \le M {{\,\mathrm{\textrm{E}}\,}}\left\{ \varphi _j(O;{\hat{\nu }}_{-k}) - \varphi _j(O;\nu )\right\} ^2 = o_p(1),$$

where the inequality follows from assumption (i) and the equality follows from lemma D2, since we have assumed that assumption B3 holds for each *k*. Consistency in $L_2(P)$ of the second term follows by analogous arguments, replacing assumption B3 with assumption (ii). Consistency of the third term in $L_2(P)$ follows from assumption (iii) if $[\varphi _j(O;\nu ) - \tau _j^l(X_{-l})]^2$ is bounded almost surely. To that end, observe that for almost all $o \in {\mathcal {O}}$ we have

$$\begin{aligned}&[\varphi _j(o;\nu ) - \tau _j^l(x_{-l})]^2 \\ \le&2\left[ \tau_j (x) - \tau _j^l(x_{-l})\right] ^2 + 4 \left[ \sum _{i=1,2}\frac{\mathbb {1}(A=1)}{\pi (1\mid x)}\int _0^{t^*} \frac{H_{ij}(s,t^*\mid 1, x)}{S_c(s\mid 1,x)}\textrm{d}M_i(s\mid ,a,x) \right] ^2 \\&+ 4\left[ \sum _{i=1,2} \frac{\mathbb {1}(A=0)}{\pi (0\mid x)}\int _0^{t^*} \frac{H_{ij}(s,t^*\mid 0, x)}{S_c(s\mid 0,x)}\textrm{d}M_i(s\mid ,0,x) \right] ^2. \end{aligned}$$

Hence consistency of the empirical process term follows, if we can bound each term in the display above. The first term is clearly bounded, since $\tau_j$ is bounded by $t^*$. For the second and third term observe that

$$\begin{aligned}&\left[ \sum _{i=1,2} \frac{\mathbb {1}(A=a)}{\pi (a\mid x)}\int _0^{t^*} \frac{H_{ij}(s,t^*\mid a, x)}{S_c(s\mid a,x)}\textrm{d}M_i(s\mid ,a,x) \right] ^2 \\ \le&2\eta ^{-2}\left[ \sum _{i=1,2} \int _0^{t^*} H_{ij}(s,t^*\mid a, x)\textrm{d}N_i(s\mid ,a,x) \right] ^2 \\&+ 2\eta ^{-2}\left[ \sum _{i=1,2} \int _0^{t^*} H_{ij}(s,t^*\mid a, x)\mathbb {1}({\tilde{T}}\ge s) \textrm{d}\varLambda _i(s\mid ,a,x) \right] ^2 \\ \le&2 \eta ^{-2}\left[ \sum _{i=1,2} 2\eta ^{-1}t^* \right] ^2 + 2\eta ^{-2}\left[ 2\eta ^{-1}t^* \sum _{i=1,2} \int _0^{t^*} \mathbb {1}({\tilde{T}}\ge s) \textrm{d}\varLambda _i(s\mid ,a,x) \right] ^2 \\ =&32 \eta ^{-4}(t^*)^2 + 16\eta ^{-4} (t^*)^2 \left[ \int _0^{t^*} \mathbb {1}({\tilde{T}}\ge s) \textrm{d}\varLambda (s\mid ,a,x) \right] ^2 \\ \le&\eta ^{-4}(t^*)^2(32 + 16 \log (\eta ^{-1})^2) \end{aligned}$$

where the first and third inequality follows from assumption B1 and the second inequality follows from (34). Thus, it follows that $\left\Vert \phi _{\varGamma _j^l}(\cdot ;{\hat{\nu }}^1_{-k}) - \phi _{\varGamma _j^l}(\cdot ;\nu ^1)\right\Vert = o_p(1)$ for each *k*.

*Remainder term*

As in Ziersen and Martinussen (2025), we consider the decomposition

39 $$\begin{aligned} P\{ \phi _{\varGamma _j^l}(\cdot ;{\hat{\nu }}^2_{-k}) - \phi _{\varGamma _j}(\cdot ;\nu ^2) \} =&E\left\{ [\varphi (O;{\hat{\nu }}_{-k}) - \varphi _j(O;\nu )][X_l - {\hat{E}}_{-k}^l(X_{-l})] \right\} \nonumber \\&- {{\,\mathrm{\textrm{E}}\,}}\left\{ [{\hat{\tau }}_{j,-k}^l(X_{-l}) - \tau _j^l(X_{-l})][X_j - {\hat{E}}_{-k}^j(X_{-l})]\right\} \nonumber \\&-{{\,\mathrm{\textrm{E}}\,}}\left\{ [{\hat{E}}_{-k}^l(X_{-j}) - E(X_l\mid X_{-l})][\varphi _j(O;\nu ) - \tau _j^l(X_{-l})] \right\} \end{aligned}$$

and we want to show that each term is $o_p(n^{-1/2})$. For the third term we note that

$$\begin{aligned}E&(\varphi _j(O;\nu ) - \tau _j^l(X_{-l})\mid A,X) \\ =&\left( \frac{\mathbb {1}(A=1)}{\pi (1\mid X)} - \frac{\mathbb {1}(A=0)}{\pi (0\mid X)}\right) \sum _{i=1,2} E\left( \int _0^{t^*} \frac{H_{ij}(s,t^*\mid A, X)}{S_C(s\mid A, X)} \textrm{d}M_i(s\mid A, X) \bigg | A, X \right) \\ =&0 \end{aligned}$$

since the integral is a martingale conditional on *A* and *X*, and hence, that the third term is equal to 0 by iterated expectation. The second term is $o_p(n^{-1/2})$ by Cauchy-Schwarz together with assumption (ii) and (iii). Following the derivations in the proof of D1, we can use iterated expectation to write the first term as

$$\begin{aligned}{{\,\mathrm{\textrm{E}}\,}}&\left\{ [X_l - {\hat{E}}_{-k}^l(X_{-l})]\sum _{a=0,1}\sum _{i=1,2} \int _0^{t^*} S(s\mid a, X) {\hat{H}}_{ij}(s, t^*\mid a, X) \right. \\&\left. \times \left( 1 - \frac{\pi (a\mid X)S_C(s\mid a, X)}{{\hat{\pi }}(a\mid X){\hat{S}}_C(s\mid a, X)} \right) \textrm{d}\left[ {\hat{\varLambda }}_i(s\mid a, X) - \varLambda _i(s\mid a, X) \right] \right\} . \end{aligned}$$

By assumption (i) this is bounded by

$$\begin{aligned}\sqrt{M}&\sum _{a=0,1}{{\,\mathrm{\textrm{E}}\,}}\left\{ \left| \sum _{i=1,2} \int _0^{t^*} S(s\mid a, X) {\hat{H}}_{ij}(s, t^*\mid a, X) \right. \right. \\&\left. \left. \times \left( 1 - \frac{\pi (a\mid X)S_C(s\mid a, X)}{{\hat{\pi }}(a\mid X){\hat{S}}_C(s\mid a, X)} \right) \textrm{d}\left[ {\hat{\varLambda }}_i(s\mid a, X) - \varLambda _i(s\mid a, X) \right] \right| \right\} \end{aligned}$$

which is $o_p(n^{-1/2})$ by assumption B2.

We have now shown that summands of the empirical process term and remainder term are $o_p(n^{-1/2})$ for each *k*, and hence it follows from Proposition 2 in Kennedy (2022) that ${\hat{\varGamma }}_j^{l,CF}$ is asymptotically linear with influence function given by ${\tilde{\psi }}_{\varGamma _j^l}$. The convergence in distribution of $\sqrt{n}({\hat{\varOmega }}_j^{l,CF} - \varOmega _j^l)$ follows from the central limit theorem together with Slutsky’s lemma. *□*

## Appendix E Additional simulation results

We conduct an additional simulation study with correlated covariates, to test the robustness of the estimator. The nuisance parameters are simulated from the same models as in Sect. 5, but with the covariates simulated from $X\sim {\mathcal {N}}(0, \varSigma )$ where $\varSigma _{ij} = 0.4 \cdot 0.5^{|i-j|}$. Otherwise, the setting is the same with the target parameters defined at $t^*=30$ and with a 1000 simulations for each sample size.

For each single simulation, four different nuisance settings are used, amounting to a total of 16000 estimates of $\nu = (\pi , \varLambda _1, \varLambda _2, \varLambda _c)$. In 56 instances one of the cumulative hazard estimators produced an infinite estimate when predicting the counterfactual outcome. These instances are removed from the results presented in the following. It should be noted that the error occurred with all four difference methods, i.e. **cor**, **corCF**, **RF** and **RFCF**.

### Appendix E.1 Average treatment effect

Under the multivariate normal distributed covariates the following parameters are approximated using Monte-Carlo simulation: $\psi _1(P_0) \approx -9.55$, $\psi _2(P_0) \approx 4.41$, $E\{L_1(0,t^*\mid 1, X)\} \approx 2.69$, $E\{L_1(0,t^*\mid 0, X)\} \approx 12.24$, $E\{L_2(0,t^*\mid 1, X)\} \approx 5.63$, $E\{L_2(0,t^*\mid 1, X)\} \approx 1.22$. We use the same nuisance estimators as in Sect. 5 and adopt the same naming conventions. Table 4 displays the results. The results are similar to those given in Table 1 for covariates with a uniform distribution; thus reaching the same conclusions as reported in Sect. 5.

**Table 4:** Results of 1000 simulations of estimators of $\psi _1$ with varying nuisance estimators, and with and without cross-fitting for sample sizes *n=250, 500, 750, 1000*

n	method	bias	SD	mean SE	coverage
250	**cor**	−0.2220	0.9959	1.0023	0.9419
	**corCF**	−0.1061	1.0426	1.0797	0.9519
	**RF**	−0.5178	0.9938	0.5636	0.7015
	**RFCF**	0.0380	1.5985	1.6012	0.9780
500	**cor**	−0.1211	0.7137	0.7110	0.9428
	**corCF**	−0.0805	0.7267	0.7357	0.9488
	**RF**	−0.4346	0.6877	0.3987	0.6653
	**RFCF**	−0.0466	0.9560	1.0027	0.9699
750	**cor**	−0.0848	0.5807	0.5807	0.9499
	**corCF**	−0.0583	0.5879	0.5938	0.9550
	**RF**	−0.4012	0.5620	0.3270	0.6313
	**RFCF**	−0.0658	0.7342	0.7792	0.9660
1000	**cor**	−0.0704	0.5041	0.5027	0.9419
	**corCF**	−0.0469	0.5075	0.5108	0.9510
	**RF**	−0.3799	0.4880	0.2841	0.6141
	**RFCF**	−0.0486	0.6175	0.6601	0.9759

The covariates are correlated and follow a multivariate normal distribution. The abbreveations of the methods are read as follows: **cor** corresponds to the nuisance parameters$\varLambda _1, \varLambda _2, \varLambda _c, \pi$estimated by correctly specified Cox and logistic regressions, and **RF** corresponds to the same parameters estimated by Random Forest. A suffix **CF** indicates that cross-fitting was employed in estimation of$\psi _j$. The tables gives the bias, empirical standard deviation (SD), mean of the estimated standard error (mean SE), and coverage

## Appendix F Best partially linear projection

The true values are approximated to $(\varOmega _1^1, \varOmega _1^2, \varOmega _1^3, \varOmega _1^4) \approx (4.507, 2.640, 0.664, 0.008)$ using Monte-Carlo simulation. When the covariates are correlated, the Monte-Carlo simulation is not as straight forward as in Sect. 5, since we need to estimate the conditional distributions $E(\tau _j(X)\mid X_{-l})$. As such, we generate $10^7$ draws from *X*, and for each draw we simulate 1000 draws from $X_l\mid X_{-l}$ to calculate the conditional mean. After that, the Monte-Carlo simulation proceeds as usual.

In Fig. 5 we see the biases of the different estimators for varying sample sizes. Largely, we see the biases decrease across the board, but now it is a bit more irregular compared to the uniform covarite setting in Sect. 5. The cross-fitted and non-cross-fitted versions of the correctly specified estimators (with GAM used for estimation of the conditional means) are still performing similarly, as in the uniform setting, and the biggest difference between the two settings are the performance of the estimators using random forest. Aside from $\varOmega _1^1$, the cross-fitted and non-cross-fitted estimators have switched places in terms of performance compared to the uniform setting. However, since the one-step estimators for $\varOmega _j^l$ are constructed from ratios of two separate estimators, the bias from each estimator can cancel each other out, and the estimator for $\varOmega _j^l$ can thus appear unbiased. Indeed, this is what we see here. Looking at the coverage in Fig. 6 it becomes clear that the estimator using random forest without cross-fitting does not perform as intended.

In Figs. 7 and 8 we see the probability of rejecting the hypotheses $H:\varOmega _1^4=0$ and $H:\varOmega _1^l$, *l=1,2,3*, respectively. As the true value of $\varOmega _1^4$ is approximated to 0.008, Fig. 7 no longer corresponds to a type-1 error, but rather, it is the power of the test of $H:\varOmega _1^4=0$. But since the true value is very close to zero, the performance of the estimators behave as in the uniform setting, where Fig. 3 indeed corresponded to the type-1 error. The power of the estimators shown in Fig. 8 display the same behavior as in the uniform setting.

In general, the conclusions from these simulation studies are the same as for the uniform setting, where the general recommendation is to use cross-fitting with data-adaptive estimators.
